# Supplementary material for: Metagenome fragment classification based on multiple motif-occurrence profiles
Source: PeerJ. 2014 Sep 4;2:e559. doi: 10.7717/peerj.559 (PMC4157293; doi:10.7717/peerj.559)
Supplement: Data S1 — We obtained reference genome sequence data file all.fna.tar.gz in December 2012 from the NCBI Microbial Genomes FTP site: ftp://ftp.ncbi.nih.gov/genomes/Bacteria/. At that time, the number of genome sequences was 2,112 (see the list of strains in the dataset below). [file peerj-02-559-s001.docx]

Acaryochloris_marina_MBIC11017_uid58167

Acetobacter_pasteurianus_IFO_3283_01_42C_uid158377

Acetobacter_pasteurianus_IFO_3283_01_uid59279

Acetobacter_pasteurianus_IFO_3283_03_uid158373

Acetobacter_pasteurianus_IFO_3283_07_uid158381

Acetobacter_pasteurianus_IFO_3283_12_uid158379

Acetobacter_pasteurianus_IFO_3283_22_uid158383

Acetobacter_pasteurianus_IFO_3283_26_uid158531

Acetobacter_pasteurianus_IFO_3283_32_uid158375

Acetobacterium_woodii_DSM_1030_uid88073

Acetohalobium_arabaticum_DSM_5501_uid51423

Acholeplasma_laidlawii_PG_8A_uid58901

Achromobacter_xylosoxidans_A8_uid59899

Acidaminococcus_fermentans_DSM_20731_uid43471

Acidaminococcus_intestini_RyC_MR95_uid74445

Acidianus_hospitalis_W1_uid66875

Acidilobus_saccharovorans_345_15_uid51395

Acidimicrobium_ferrooxidans_DSM_10331_uid59215

Acidiphilium_cryptum_JF_5_uid58447

Acidiphilium_multivorum_AIU301_uid63345

Acidithiobacillus_caldus_SM_1_uid70791

Acidithiobacillus_ferrivorans_SS3_uid67387

Acidithiobacillus_ferrooxidans_ATCC_23270_uid57649

Acidithiobacillus_ferrooxidans_ATCC_53993_uid58613

Acidobacterium_MP5ACTX9_uid50551

Acidobacterium_capsulatum_ATCC_51196_uid59127

Acidothermus_cellulolyticus_11B_uid58501

Acidovorax_JS42_uid58427

Acidovorax_avenae_ATCC_19860_uid42497

Acidovorax_citrulli_AAC00_1_uid58429

Acidovorax_ebreus_TPSY_uid59233

Aciduliprofundum_boonei_T469_uid43333

Acinetobacter_ADP1_uid61597

Acinetobacter_baumannii_1656_2_uid158677

Acinetobacter_baumannii_AB0057_uid59083

Acinetobacter_baumannii_AB307_0294_uid59271

Acinetobacter_baumannii_ACICU_uid58765

Acinetobacter_baumannii_ATCC_17978_uid58731

Acinetobacter_baumannii_AYE_uid61637

Acinetobacter_baumannii_MDR_TJ_uid162739

Acinetobacter_baumannii_MDR_ZJ06_uid158685

Acinetobacter_baumannii_SDF_uid61601

Acinetobacter_baumannii_TCDC_AB0715_uid158679

Acinetobacter_baumannii_TYTH_1_uid176498

Acinetobacter_calcoaceticus_PHEA_2_uid83123

Acinetobacter_oleivorans_DR1_uid50119

Actinobacillus_pleuropneumoniae_serovar_3_JL03_uid58891

Actinobacillus_pleuropneumoniae_serovar_5b_L20_uid58789

Actinobacillus_pleuropneumoniae_serovar_7_AP76_uid59231

Actinobacillus_succinogenes_130Z_uid58247

Actinobacillus_suis_H91_0380_uid176363

Actinoplanes_SE50_110_uid162333

Actinoplanes_missouriensis_431_uid158169

Actinosynnema_mirum_DSM_43827_uid58951

Advenella_kashmirensis_WT001_uid80859

Aequorivita_sublithincola_DSM_14238_uid168181

Aerococcus_urinae_ACS_120_V_Col10a_uid64757

Aeromonas_hydrophila_ATCC_7966_uid58617

Aeromonas_salmonicida_A449_uid58631

Aeromonas_veronii_B565_uid66323

Aeropyrum_pernix_K1_uid57757

Aggregatibacter_actinomycetemcomitans_ANH9381_uid80743

Aggregatibacter_actinomycetemcomitans_D11S_1_uid41333

Aggregatibacter_actinomycetemcomitans_D7S_1_uid46989

Aggregatibacter_aphrophilus_NJ8700_uid59407

Agrobacterium_H13_3_uid63403

Agrobacterium_radiobacter_K84_uid58269

Agrobacterium_tumefaciens_C58_uid57865

Agrobacterium_vitis_S4_uid58249

Akkermansia_muciniphila_ATCC_BAA_835_uid58985

Alcanivorax_borkumensis_SK2_uid58169

Alcanivorax_dieselolei_B5_uid176364

Alicycliphilus_denitrificans_BC_uid49953

Alicycliphilus_denitrificans_K601_uid66307

Alicyclobacillus_acidocaldarius_DSM_446_uid59199

Alicyclobacillus_acidocaldarius_Tc_4_1_uid158681

Aliivibrio_salmonicida_LFI1238_uid59251

Alistipes_finegoldii_DSM_17242_uid168180

Alkalilimnicola_ehrlichii_MLHE_1_uid58467

Alkaliphilus_metalliredigens_QYMF_uid58171

Alkaliphilus_oremlandii_OhILAs_uid58495

Allochromatium_vinosum_DSM_180_uid46083

Alteromonas_SN2_uid67349

Alteromonas_macleodii_ATCC_27126_uid55253

Alteromonas_macleodii__Balearic_Sea_AD45__uid176366

Alteromonas_macleodii__Black_Sea_11__uid176365

Alteromonas_macleodii__Deep_ecotype__uid58251

Alteromonas_macleodii__English_Channel_673__uid176367

Aminobacterium_colombiense_DSM_12261_uid47083

Ammonifex_degensii_KC4_uid41053

Amphibacillus_xylanus_NBRC_15112_uid176453

Amycolatopsis_mediterranei_S699_uid158689

Amycolatopsis_mediterranei_S699_uid171830

Amycolatopsis_mediterranei_U32_uid50565

Amycolicicoccus_subflavus_DQS3_9A1_uid67253

Anabaena_variabilis_ATCC_29413_uid58043

Anaerobaculum_mobile_DSM_13181_uid168323

Anaerococcus_prevotii_DSM_20548_uid59219

Anaerolinea_thermophila_UNI_1_uid62245

Anaeromyxobacter_Fw109_5_uid58755

Anaeromyxobacter_K_uid58953

Anaeromyxobacter_dehalogenans_2CP_1_uid58989

Anaeromyxobacter_dehalogenans_2CP_C_uid58135

Anaplasma_centrale_Israel_uid42155

Anaplasma_marginale_Florida_uid58577

Anaplasma_marginale_Maries_uid57629

Anaplasma_phagocytophilum_HZ_uid57951

Anoxybacillus_flavithermus_WK1_uid59135

Aquifex_aeolicus_VF5_uid57765

Arcanobacterium_haemolyticum_DSM_20595_uid49489

Archaeoglobus_fulgidus_DSM_4304_uid57717

Archaeoglobus_profundus_DSM_5631_uid43493

Archaeoglobus_veneficus_SNP6_uid65269

Arcobacter_L_uid158135

Arcobacter_butzleri_ED_1_uid158699

Arcobacter_butzleri_RM4018_uid58557

Arcobacter_nitrofigilis_DSM_7299_uid49001

Aromatoleum_aromaticum_EbN1_uid58231

Arthrobacter_FB24_uid58141

Arthrobacter_arilaitensis_Re117_uid53509

Arthrobacter_aurescens_TC1_uid58109

Arthrobacter_chlorophenolicus_A6_uid58969

Arthrobacter_nitroguajacolicus_Rue61a_uid174511

Arthrobacter_phenanthrenivorans_Sphe3_uid63629

Aster_yellows_witches_broom_phytoplasma_AYWB_uid58297

Asticcacaulis_excentricus_CB_48_uid55641

Atopobium_parvulum_DSM_20469_uid59195

Azoarcus_BH72_uid61603

Azorhizobium_caulinodans_ORS_571_uid58905

Azospirillum_B510_uid46085

Azospirillum_brasilense_Sp245_uid162161

Azospirillum_lipoferum_4B_uid82343

Azotobacter_vinelandii_DJ_uid57597

Bacillus_JS_uid162189

Bacillus_amyloliquefaciens_DSM_7_uid53535

Bacillus_amyloliquefaciens_FZB42_uid58271

Bacillus_amyloliquefaciens_LL3_uid158133

Bacillus_amyloliquefaciens_TA208_uid158701

Bacillus_amyloliquefaciens_XH7_uid158881

Bacillus_amyloliquefaciens_Y2_uid165195

Bacillus_amyloliquefaciens_plantarum_CAU_B946_uid84215

Bacillus_amyloliquefaciens_plantarum_YAU_B9601_Y2_uid159001

Bacillus_anthracis_A0248_uid59385

Bacillus_anthracis_Ames_uid57909

Bacillus_anthracis_CDC_684_uid59303

Bacillus_anthracis_H9401_uid162021

Bacillus_anthracis_Sterne_uid58091

Bacillus_anthracis__Ames_Ancestor__uid58083

Bacillus_atrophaeus_1942_uid59887

Bacillus_cellulosilyticus_DSM_2522_uid43329

Bacillus_cereus_03BB102_uid59299

Bacillus_cereus_AH187_uid58753

Bacillus_cereus_AH820_uid58751

Bacillus_cereus_ATCC_10987_uid57673

Bacillus_cereus_ATCC_14579_uid57975

Bacillus_cereus_B4264_uid58757

Bacillus_cereus_E33L_uid58103

Bacillus_cereus_F837_76_uid83611

Bacillus_cereus_G9842_uid58759

Bacillus_cereus_NC7401_uid82815

Bacillus_cereus_Q1_uid58529

Bacillus_cereus_biovar_anthracis_CI_uid50615

Bacillus_clausii_KSM_K16_uid58237

Bacillus_coagulans_2_6_uid68053

Bacillus_coagulans_36D1_uid54335

Bacillus_cytotoxicus_NVH_391_98_uid58317

Bacillus_halodurans_C_125_uid57791

Bacillus_licheniformis_ATCC_14580_uid58097

Bacillus_licheniformis_DSM_13___ATCC_14580_uid58199

Bacillus_megaterium_DSM319_uid48371

Bacillus_megaterium_QM_B1551_uid15862

Bacillus_megaterium_WSH_002_uid159841

Bacillus_pseudofirmus_OF4_uid45847

Bacillus_pumilus_SAFR_032_uid59017

Bacillus_selenitireducens_MLS10_uid49513

Bacillus_subtilis_168_uid57675

Bacillus_subtilis_BSn5_uid62463

Bacillus_subtilis_RO_NN_1_uid158879

Bacillus_subtilis_spizizenii_TU_B_10_uid73967

Bacillus_subtilis_spizizenii_W23_uid51879

Bacillus_thuringiensis_Al_Hakam_uid58795

Bacillus_thuringiensis_BMB171_uid49135

Bacillus_thuringiensis_Bt407_uid177931

Bacillus_thuringiensis_MC28_uid176369

Bacillus_thuringiensis_serovar_chinensis_CT_43_uid158151

Bacillus_thuringiensis_serovar_finitimus_YBT_020_uid158875

Bacillus_thuringiensis_serovar_konkukian_97_27_uid58089

Bacillus_weihenstephanensis_KBAB4_uid58315

Bacteriovorax_marinus_SJ_uid82341

Bacteroides_fragilis_638R_uid84217

Bacteroides_fragilis_NCTC_9343_uid57639

Bacteroides_fragilis_YCH46_uid58195

Bacteroides_helcogenes_P_36_108_uid62135

Bacteroides_salanitronis_DSM_18170_uid63269

Bacteroides_thetaiotaomicron_VPI_5482_uid62913

Bacteroides_vulgatus_ATCC_8482_uid58253

Bartonella_bacilliformis_KC583_uid58533

Bartonella_clarridgeiae_73_uid62131

Bartonella_grahamii_as4aup_uid59405

Bartonella_henselae_Houston_1_uid57745

Bartonella_quintana_Toulouse_uid57635

Bartonella_tribocorum_CIP_105476_uid59129

Baumannia_cicadellinicola_Hc__Homalodisca_coagulata__uid58111

Bdellovibrio_bacteriovorus_HD100_uid61595

Beijerinckia_indica_ATCC_9039_uid59057

Belliella_baltica_DSM_15883_uid168182

Beutenbergia_cavernae_DSM_12333_uid59047

Bifidobacterium_adolescentis_ATCC_15703_uid58559

Bifidobacterium_animalis_ATCC_25527_uid162513

Bifidobacterium_animalis_lactis_AD011_uid58911

Bifidobacterium_animalis_lactis_B420_uid163691

Bifidobacterium_animalis_lactis_BB_12_uid158871

Bifidobacterium_animalis_lactis_BLC1_uid158867

Bifidobacterium_animalis_lactis_Bi_07_uid163693

Bifidobacterium_animalis_lactis_Bl_04_uid59359

Bifidobacterium_animalis_lactis_CNCM_I_2494_uid158869

Bifidobacterium_animalis_lactis_DSM_10140_uid59357

Bifidobacterium_animalis_lactis_V9_uid158865

Bifidobacterium_asteroides_PRL2011_uid176921

Bifidobacterium_bifidum_BGN4_uid167988

Bifidobacterium_bifidum_PRL2010_uid59883

Bifidobacterium_bifidum_S17_uid59545

Bifidobacterium_breve_ACS_071_V_Sch8b_uid158863

Bifidobacterium_dentium_Bd1_uid43091

Bifidobacterium_longum_BBMN68_uid60163

Bifidobacterium_longum_DJO10A_uid58833

Bifidobacterium_longum_JCM_1217_uid62695

Bifidobacterium_longum_JDM301_uid49131

Bifidobacterium_longum_KACC_91563_uid158861

Bifidobacterium_longum_NCC2705_uid57939

Bifidobacterium_longum_infantis_157F_uid62693

Bifidobacterium_longum_infantis_ATCC_15697_uid159865

Bifidobacterium_longum_infantis_ATCC_15697_uid58677

Blastococcus_saxobsidens_DD2_uid89391

Blattabacterium__Blaberus_giganteus__uid165873

Blattabacterium__Blattella_germanica__Bge_uid41533

Blattabacterium__Cryptocercus_punctulatus__Cpu_uid81083

Blattabacterium__Mastotermes_darwiniensis__MADAR_uid77127

Blattabacterium__Periplaneta_americana__BPLAN_uid41287

Bordetella_avium_197N_uid61563

Bordetella_bronchiseptica_RB50_uid57613

Bordetella_parapertussis_12822_uid57615

Bordetella_pertussis_CS_uid158859

Bordetella_pertussis_Tohama_I_uid57617

Bordetella_petrii_DSM_12804_uid61631

Borrelia_afzelii_PKo_uid159867

Borrelia_afzelii_PKo_uid58653

Borrelia_bissettii_DN127_uid71231

Borrelia_burgdorferi_B31_uid57581

Borrelia_burgdorferi_JD1_uid161197

Borrelia_burgdorferi_N40_uid161241

Borrelia_burgdorferi_ZS7_uid59429

Borrelia_crocidurae_Achema_uid162335

Borrelia_duttonii_Ly_uid58791

Borrelia_garinii_BgVir_uid162165

Borrelia_garinii_PBi_uid58125

Borrelia_hermsii_DAH_uid59225

Borrelia_recurrentis_A1_uid58793

Borrelia_turicatae_91E135_uid58311

Brachybacterium_faecium_DSM_4810_uid58649

Brachyspira_hyodysenteriae_WA1_uid59291

Brachyspira_intermedia_PWS_A_uid158369

Brachyspira_murdochii_DSM_12563_uid48819

Brachyspira_pilosicoli_95_1000_uid50609

Brachyspira_pilosicoli_B2904_uid175255

Bradyrhizobium_BTAi1_uid58505

Bradyrhizobium_ORS_278_uid58941

Bradyrhizobium_S23321_uid158167

Bradyrhizobium_japonicum_USDA_110_uid57599

Bradyrhizobium_japonicum_USDA_6_uid158851

Brevibacillus_brevis_NBRC_100599_uid59175

Brevundimonas_subvibrioides_ATCC_15264_uid42117

Brucella_abortus_A13334_uid83615

Brucella_abortus_S19_uid58873

Brucella_abortus_bv__1_9_941_uid58019

Brucella_canis_ATCC_23365_uid59009

Brucella_canis_HSK_A52141_uid83613

Brucella_melitensis_ATCC_23457_uid59241

Brucella_melitensis_M28_uid158857

Brucella_melitensis_M5_90_uid158855

Brucella_melitensis_NI_uid158853

Brucella_melitensis_biovar_Abortus_2308_uid62937

Brucella_melitensis_bv__1_16M_uid57735

Brucella_microti_CCM_4915_uid59319

Brucella_ovis_ATCC_25840_uid58113

Brucella_pinnipedialis_B2_94_uid71131

Brucella_suis_1330_uid159871

Brucella_suis_1330_uid57927

Brucella_suis_ATCC_23445_uid59015

Brucella_suis_VBI22_uid83617

Buchnera_aphidicola_5A__Acyrthosiphon_pisum__uid59285

Buchnera_aphidicola_APS__Acyrthosiphon_pisum__uid57805

Buchnera_aphidicola_Ak__Acyrthosiphon_kondoi__uid158533

Buchnera_aphidicola_Bp__Baizongia_pistaciae__uid57827

Buchnera_aphidicola_Cc__Cinara_cedri__uid58579

Buchnera_aphidicola_JF98__Acyrthosiphon_pisum__uid158845

Buchnera_aphidicola_JF99__Acyrthosiphon_pisum__uid158847

Buchnera_aphidicola_LL01__Acyrthosiphon_pisum__uid158843

Buchnera_aphidicola_Sg__Schizaphis_graminum__uid57913

Buchnera_aphidicola_TLW03__Acyrthosiphon_pisum__uid158849

Buchnera_aphidicola_Tuc7__Acyrthosiphon_pisum__uid59283

Buchnera_aphidicola_Ua__Uroleucon_ambrosiae__uid158535

Buchnera_aphidicola__Cinara_tujafilina__uid68101

Burkholderia_383_uid58073

Burkholderia_CCGE1001_uid42975

Burkholderia_CCGE1002_uid42523

Burkholderia_CCGE1003_uid46253

Burkholderia_KJ006_uid165871

Burkholderia_YI23_uid81081

Burkholderia_ambifaria_AMMD_uid58303

Burkholderia_ambifaria_MC40_6_uid58701

Burkholderia_cenocepacia_AU_1054_uid58371

Burkholderia_cenocepacia_HI2424_uid58369

Burkholderia_cenocepacia_J2315_uid57953

Burkholderia_cenocepacia_MC0_3_uid58769

Burkholderia_cepacia_GG4_uid173858

Burkholderia_gladioli_BSR3_uid66301

Burkholderia_glumae_BGR1_uid59397

Burkholderia_mallei_ATCC_23344_uid57725

Burkholderia_mallei_NCTC_10229_uid58383

Burkholderia_mallei_NCTC_10247_uid58385

Burkholderia_mallei_SAVP1_uid58387

Burkholderia_multivorans_ATCC_17616_uid58697

Burkholderia_multivorans_ATCC_17616_uid58909

Burkholderia_phymatum_STM815_uid58699

Burkholderia_phytofirmans_PsJN_uid58729

Burkholderia_pseudomallei_1026b_uid162511

Burkholderia_pseudomallei_1106a_uid58515

Burkholderia_pseudomallei_1710b_uid58391

Burkholderia_pseudomallei_668_uid58389

Burkholderia_pseudomallei_K96243_uid57733

Burkholderia_pseudomallei_MSHR346_uid29855

Burkholderia_rhizoxinica_HKI_454_uid60487

Burkholderia_thailandensis_E264_uid58081

Burkholderia_vietnamiensis_G4_uid58075

Burkholderia_xenovorans_LB400_uid57823

Butyrivibrio_proteoclasticus_B316_uid51489

Caldicellulosiruptor_bescii_DSM_6725_uid59201

Caldicellulosiruptor_hydrothermalis_108_uid60157

Caldicellulosiruptor_kristjanssonii_177R1B_uid60393

Caldicellulosiruptor_kronotskyensis_2002_uid60491

Caldicellulosiruptor_lactoaceticus_6A_uid60575

Caldicellulosiruptor_obsidiansis_OB47_uid51501

Caldicellulosiruptor_owensensis_OL_uid60165

Caldicellulosiruptor_saccharolyticus_DSM_8903_uid58289

Caldilinea_aerophila_DSM_14535___NBRC_104270_uid158165

Caldisericum_exile_AZM16c01_uid158173

Calditerrivibrio_nitroreducens_DSM_19672_uid60821

Caldivirga_maquilingensis_IC_167_uid58711

Campylobacter_concisus_13826_uid58667

Campylobacter_curvus_525_92_uid58669

Campylobacter_fetus_82_40_uid58545

Campylobacter_hominis_ATCC_BAA_381_uid58981

Campylobacter_jejuni_81116_uid58771

Campylobacter_jejuni_81_176_uid58503

Campylobacter_jejuni_IA3902_uid159531

Campylobacter_jejuni_ICDCCJ07001_uid61249

Campylobacter_jejuni_M1_uid159535

Campylobacter_jejuni_NCTC_11168___ATCC_700819_uid57587

Campylobacter_jejuni_PT14_uid176499

Campylobacter_jejuni_RM1221_uid57899

Campylobacter_jejuni_S3_uid159533

Campylobacter_jejuni_doylei_269_97_uid58671

Campylobacter_lari_RM2100_uid58115

Candidatus_Accumulibacter_phosphatis_clade_IIA_UW_1_uid59207

Candidatus_Amoebophilus_asiaticus_5a2_uid58963

Candidatus_Arthromitus_SFB_mouse_Japan_uid71379

Candidatus_Arthromitus_SFB_mouse_Yit_uid159517

Candidatus_Arthromitus_SFB_rat_Yit_uid73425

Candidatus_Azobacteroides_pseudotrichonymphae_genomovar__CFP2_uid59163

Candidatus_Blochmannia_floridanus_uid57999

Candidatus_Blochmannia_pennsylvanicus_BPEN_uid58329

Candidatus_Blochmannia_vafer_BVAF_uid62083

Candidatus_Cardinium_hertigii_uid175524

Candidatus_Carsonella_ruddii_CE_isolate_Thao2000_uid172732

Candidatus_Carsonella_ruddii_CS_isolate_Thao2000_uid172733

Candidatus_Carsonella_ruddii_HC_isolate_Thao2000_uid172734

Candidatus_Carsonella_ruddii_HT_isolate_Thao2000_uid172735

Candidatus_Carsonella_ruddii_PC_isolate_NHV_uid172736

Candidatus_Carsonella_ruddii_uid58773

Candidatus_Chloracidobacterium_thermophilum_B_uid73587

Candidatus_Cloacamonas_acidaminovorans_uid62959

Candidatus_Desulforudis_audaxviator_MP104C_uid59067

Candidatus_Hamiltonella_defensa_5AT__Acyrthosiphon_pisum__uid59289

Candidatus_Hodgkinia_cicadicola_Dsem_uid59311

Candidatus_Korarchaeum_cryptofilum_OPF8_uid58601

Candidatus_Koribacter_versatilis_Ellin345_uid58479

Candidatus_Liberibacter_asiaticus_psy62_uid59227

Candidatus_Liberibacter_solanacearum_CLso_ZC1_uid61245

Candidatus_Methylomirabilis_oxyfera_uid161981

Candidatus_Midichloria_mitochondrii_IricVA_uid68687

Candidatus_Moranella_endobia_PCIT_uid68739

Candidatus_Nitrospira_defluvii_uid51175

Candidatus_Pelagibacter_IMCC9063_uid66305

Candidatus_Pelagibacter_ubique_HTCC1062_uid58401

Candidatus_Phytoplasma_australiense_uid61641

Candidatus_Phytoplasma_mali_uid59087

Candidatus_Portiera_aleyrodidarum_BT_B_uid173859

Candidatus_Portiera_aleyrodidarum_BT_B_uid176373

Candidatus_Protochlamydia_amoebophila_UWE25_uid58079

Candidatus_Puniceispirillum_marinum_IMCC1322_uid47081

Candidatus_Rickettsia_amblyommii_GAT_30V_uid156845

Candidatus_Riesia_pediculicola_USDA_uid46841

Candidatus_Ruthia_magnifica_Cm__Calyptogena_magnifica__uid58645

Candidatus_Solibacter_usitatus_Ellin6076_uid58139

Candidatus_Sulcia_muelleri_CARI_uid52535

Candidatus_Sulcia_muelleri_DMIN_uid47075

Candidatus_Sulcia_muelleri_GWSS_uid58943

Candidatus_Sulcia_muelleri_SMDSEM_uid59393

Candidatus_Tremblaya_princeps_PCIT_uid68741

Candidatus_Tremblaya_princeps_PCVAL_uid159519

Candidatus_Vesicomyosocius_okutanii_HA_uid59427

Candidatus_Zinderia_insecticola_CARI_uid52459

Capnocytophaga_canimorsus_Cc5_uid70727

Capnocytophaga_ochracea_DSM_7271_uid59197

Carboxydothermus_hydrogenoformans_Z_2901_uid57821

Carnobacterium_17_4_uid65789

Catenulispora_acidiphila_DSM_44928_uid59077

Caulobacter_K31_uid58551

Caulobacter_crescentus_CB15_uid57891

Caulobacter_crescentus_NA1000_uid59307

Caulobacter_segnis_ATCC_21756_uid41709

Cellulomonas_fimi_ATCC_484_uid66779

Cellulomonas_flavigena_DSM_20109_uid48821

Cellulophaga_algicola_DSM_14237_uid62159

Cellulophaga_lytica_DSM_7489_uid63401

Cellvibrio_japonicus_Ueda107_uid59139

Cenarchaeum_symbiosum_A_uid61411

Chelativorans_BNC1_uid58069

Chitinophaga_pinensis_DSM_2588_uid59113

Chlamydia_muridarum_Nigg_uid57785

Chlamydia_trachomatis_434_Bu_uid61633

Chlamydia_trachomatis_A2497_uid159863

Chlamydia_trachomatis_A2497_uid159993

Chlamydia_trachomatis_A_HAR_13_uid58333

Chlamydia_trachomatis_B_Jali20_OT_uid59351

Chlamydia_trachomatis_B_TZ1A828_OT_uid59349

Chlamydia_trachomatis_D_EC_uid159881

Chlamydia_trachomatis_D_LC_uid159879

Chlamydia_trachomatis_D_UW_3_CX_uid57637

Chlamydia_trachomatis_E_11023_uid161369

Chlamydia_trachomatis_E_150_uid161403

Chlamydia_trachomatis_E_SW3_uid167483

Chlamydia_trachomatis_F_SW4_uid167484

Chlamydia_trachomatis_F_SW5_uid167485

Chlamydia_trachomatis_G_11074_uid161409

Chlamydia_trachomatis_G_11222_uid161361

Chlamydia_trachomatis_G_9301_uid161377

Chlamydia_trachomatis_G_9768_uid161353

Chlamydia_trachomatis_L2b_UCH_1_proctitis_uid61635

Chlamydia_trachomatis_L2c_uid68843

Chlamydia_trachomatis_Sweden2_uid161995

Chlamydophila_abortus_S26_3_uid57963

Chlamydophila_caviae_GPIC_uid57783

Chlamydophila_felis_Fe_C_56_uid57971

Chlamydophila_pecorum_E58_uid66295

Chlamydophila_pneumoniae_AR39_uid57809

Chlamydophila_pneumoniae_CWL029_uid57811

Chlamydophila_pneumoniae_J138_uid57829

Chlamydophila_pneumoniae_LPCoLN_uid159529

Chlamydophila_pneumoniae_TW_183_uid57997

Chlamydophila_psittaci_01DC11_uid159527

Chlamydophila_psittaci_02DC15_uid159521

Chlamydophila_psittaci_08DC60_uid159525

Chlamydophila_psittaci_6BC_uid159845

Chlamydophila_psittaci_6BC_uid63621

Chlamydophila_psittaci_C19_98_uid159523

Chlamydophila_psittaci_CP3_uid175578

Chlamydophila_psittaci_NJ1_uid175579

Chlamydophila_psittaci_RD1_uid162063

Chlorobaculum_parvum_NCIB_8327_uid59185

Chlorobium_chlorochromatii_CaD3_uid58375

Chlorobium_limicola_DSM_245_uid58127

Chlorobium_luteolum_DSM_273_uid58175

Chlorobium_phaeobacteroides_BS1_uid58131

Chlorobium_phaeobacteroides_DSM_266_uid58133

Chlorobium_phaeovibrioides_DSM_265_uid58129

Chlorobium_tepidum_TLS_uid57897

Chloroflexus_Y_400_fl_uid59085

Chloroflexus_aggregans_DSM_9485_uid58621

Chloroflexus_aurantiacus_J_10_fl_uid57657

Chloroherpeton_thalassium_ATCC_35110_uid59187

Chromobacterium_violaceum_ATCC_12472_uid58001

Chromohalobacter_salexigens_DSM_3043_uid62921

Citrobacter_koseri_ATCC_BAA_895_uid58143

Citrobacter_rodentium_ICC168_uid43089

Clavibacter_michiganensis_NCPPB_382_uid61625

Clavibacter_michiganensis_sepedonicus_uid61577

Clostridiales_genomosp__BVAB3_UPII9_5_uid46219

Clostridium_BNL1100_uid84307

Clostridium_SY8519_uid68705

Clostridium_acetobutylicum_ATCC_824_uid57677

Clostridium_acetobutylicum_DSM_1731_uid68293

Clostridium_acetobutylicum_EA_2018_uid159515

Clostridium_acidurici_9a_uid176126

Clostridium_beijerinckii_NCIMB_8052_uid58137

Clostridium_botulinum_A2_Kyoto_uid59229

Clostridium_botulinum_A3_Loch_Maree_uid59149

Clostridium_botulinum_A_ATCC_19397_uid58927

Clostridium_botulinum_A_ATCC_3502_uid61579

Clostridium_botulinum_A_Hall_uid58931

Clostridium_botulinum_B1_Okra_uid59147

Clostridium_botulinum_BKT015925_uid66203

Clostridium_botulinum_B_Eklund_17B_uid176095

Clostridium_botulinum_B_Eklund_17B_uid59159

Clostridium_botulinum_Ba4_657_uid59173

Clostridium_botulinum_E3_Alaska_E43_uid59157

Clostridium_botulinum_F_230613_uid159513

Clostridium_botulinum_F_Langeland_uid58929

Clostridium_botulinum_H04402_065_uid162091

Clostridium_cellulolyticum_H10_uid58709

Clostridium_cellulovorans_743B_uid51503

Clostridium_clariflavum_DSM_19732_uid82345

Clostridium_difficile_2007855_uid158365

Clostridium_difficile_630_uid57679

Clostridium_difficile_BI1_uid158363

Clostridium_difficile_CD196_uid41017

Clostridium_difficile_CF5_uid158359

Clostridium_difficile_M120_uid158361

Clostridium_difficile_R20291_uid40921

Clostridium_kluyveri_DSM_555_uid58885

Clostridium_kluyveri_NBRC_12016_uid59369

Clostridium_lentocellum_DSM_5427_uid49117

Clostridium_ljungdahlii_DSM_13528_uid50583

Clostridium_novyi_NT_uid58643

Clostridium_perfringens_13_uid57681

Clostridium_perfringens_ATCC_13124_uid57901

Clostridium_perfringens_SM101_uid58117

Clostridium_phytofermentans_ISDg_uid58519

Clostridium_saccharolyticum_WM1_uid51419

Clostridium_sticklandii_DSM_519_uid59585

Clostridium_tetani_E88_uid57683

Clostridium_thermocellum_ATCC_27405_uid57917

Clostridium_thermocellum_DSM_1313_uid161989

Collimonas_fungivorans_Ter331_uid70793

Colwellia_psychrerythraea_34H_uid57855

Comamonas_testosteroni_CNB_2_uid62961

Conexibacter_woesei_DSM_14684_uid43467

Coprothermobacter_proteolyticus_DSM_5265_uid59253

Coraliomargarita_akajimensis_DSM_45221_uid47079

Corallococcus_coralloides_DSM_2259_uid157997

Coriobacterium_glomerans_PW2_uid65787

Corynebacterium_aurimucosum_ATCC_700975_uid59409

Corynebacterium_diphtheriae_241_uid83607

Corynebacterium_diphtheriae_31A_uid84309

Corynebacterium_diphtheriae_BH8_uid84311

Corynebacterium_diphtheriae_C7__beta__uid84313

Corynebacterium_diphtheriae_CDCE_8392_uid84295

Corynebacterium_diphtheriae_HC01_uid84297

Corynebacterium_diphtheriae_HC02_uid84317

Corynebacterium_diphtheriae_HC03_uid84299

Corynebacterium_diphtheriae_HC04_uid84301

Corynebacterium_diphtheriae_INCA_402_uid83605

Corynebacterium_diphtheriae_NCTC_13129_uid57691

Corynebacterium_diphtheriae_PW8_uid84303

Corynebacterium_diphtheriae_VA01_uid84305

Corynebacterium_efficiens_YS_314_uid62905

Corynebacterium_glutamicum_ATCC_13032_uid57905

Corynebacterium_glutamicum_ATCC_13032_uid61611

Corynebacterium_glutamicum_R_uid58897

Corynebacterium_jeikeium_K411_uid58399

Corynebacterium_kroppenstedtii_DSM_44385_uid59411

Corynebacterium_pseudotuberculosis_1002_uid159677

Corynebacterium_pseudotuberculosis_1_06_A_uid159665

Corynebacterium_pseudotuberculosis_258_uid167260

Corynebacterium_pseudotuberculosis_267_uid162175

Corynebacterium_pseudotuberculosis_316_uid89381

Corynebacterium_pseudotuberculosis_31_uid162167

Corynebacterium_pseudotuberculosis_3_99_5_uid83609

Corynebacterium_pseudotuberculosis_42_02_A_uid159669

Corynebacterium_pseudotuberculosis_C231_uid159675

Corynebacterium_pseudotuberculosis_CIP_52_97_uid159667

Corynebacterium_pseudotuberculosis_Cp162_uid168258

Corynebacterium_pseudotuberculosis_FRC41_uid50585

Corynebacterium_pseudotuberculosis_I19_uid159673

Corynebacterium_pseudotuberculosis_P54B96_uid157909

Corynebacterium_pseudotuberculosis_PAT10_uid159671

Corynebacterium_resistens_DSM_45100_uid50555

Corynebacterium_ulcerans_0102_uid169879

Corynebacterium_ulcerans_809_uid159659

Corynebacterium_ulcerans_BR_AD22_uid68291

Corynebacterium_urealyticum_DSM_7109_uid61639

Corynebacterium_variabile_DSM_44702_uid62003

Coxiella_burnetii_CbuG_Q212_uid58893

Coxiella_burnetii_CbuK_Q154_uid58895

Coxiella_burnetii_Dugway_5J108_111_uid58629

Coxiella_burnetii_RSA_331_uid58637

Coxiella_burnetii_RSA_493_uid57631

Croceibacter_atlanticus_HTCC2559_uid49661

Cronobacter_sakazakii_ATCC_BAA_894_uid58145

Cronobacter_sakazakii_ES15_uid167045

Cronobacter_turicensis_z3032_uid40821

Cryptobacterium_curtum_DSM_15641_uid59041

Cupriavidus_metallidurans_CH34_uid57815

Cupriavidus_necator_N_1_uid68689

Cupriavidus_taiwanensis_LMG_19424_uid61615

Cyanothece_ATCC_51142_uid59013

Cyanothece_PCC_7424_uid59025

Cyanothece_PCC_7425_uid59435

Cyanothece_PCC_7822_uid52547

Cyanothece_PCC_8801_uid59027

Cyanothece_PCC_8802_uid59143

Cyclobacterium_marinum_DSM_745_uid71485

Cycloclasticus_P1_uid176368

Cytophaga_hutchinsonii_ATCC_33406_uid57651

Dechloromonas_aromatica_RCB_uid58025

Dechlorosoma_suillum_PS_uid81439

Deferribacter_desulfuricans_SSM1_uid46653

Dehalobacter_11DCA_uid177715

Dehalobacter_CF_uid177714

Dehalococcoides_BAV1_uid58477

Dehalococcoides_CBDB1_uid58413

Dehalococcoides_GT_uid42115

Dehalococcoides_VS_uid42393

Dehalococcoides_ethenogenes_195_uid57763

Dehalogenimonas_lykanthroporepellens_BL_DC_9_uid48131

Deinococcus_deserti_VCD115_uid58615

Deinococcus_geothermalis_DSM_11300_uid58275

Deinococcus_gobiensis_I_0_uid162509

Deinococcus_maricopensis_DSM_21211_uid62225

Deinococcus_proteolyticus_MRP_uid63399

Deinococcus_radiodurans_R1_uid57665

Delftia_Cs1_4_uid67319

Delftia_acidovorans_SPH_1_uid58703

Denitrovibrio_acetiphilus_DSM_12809_uid46657

Desulfarculus_baarsii_DSM_2075_uid51371

Desulfatibacillum_alkenivorans_AK_01_uid58913

Desulfitobacterium_dehalogenans_ATCC_51507_uid82553

Desulfitobacterium_hafniense_DCB_2_uid57749

Desulfitobacterium_hafniense_Y51_uid58605

Desulfobacca_acetoxidans_DSM_11109_uid65785

Desulfobacterium_autotrophicum_HRM2_uid59061

Desulfobacula_toluolica_Tol2_uid175777

Desulfobulbus_propionicus_DSM_2032_uid62265

Desulfococcus_oleovorans_Hxd3_uid58777

Desulfohalobium_retbaense_DSM_5692_uid59183

Desulfomicrobium_baculatum_DSM_4028_uid59217

Desulfomonile_tiedjei_DSM_6799_uid168320

Desulfosporosinus_acidiphilus_SJ4_uid156759

Desulfosporosinus_meridiei_DSM_13257_uid75097

Desulfosporosinus_orientis_DSM_765_uid82939

Desulfotalea_psychrophila_LSv54_uid58153

Desulfotomaculum_acetoxidans_DSM_771_uid59109

Desulfotomaculum_carboxydivorans_CO_1_SRB_uid67317

Desulfotomaculum_kuznetsovii_DSM_6115_uid67357

Desulfotomaculum_reducens_MI_1_uid58277

Desulfotomaculum_ruminis_DSM_2154_uid67507

Desulfovibrio_aespoeensis_Aspo_2_uid42613

Desulfovibrio_africanus_Walvis_Bay_uid66847

Desulfovibrio_alaskensis_G20_uid57941

Desulfovibrio_desulfuricans_ATCC_27774_uid59213

Desulfovibrio_desulfuricans_ND132_uid63159

Desulfovibrio_magneticus_RS_1_uid59309

Desulfovibrio_salexigens_DSM_2638_uid59223

Desulfovibrio_vulgaris_DP4_uid58679

Desulfovibrio_vulgaris_Hildenborough_uid57645

Desulfovibrio_vulgaris_RCH1_uid161961

Desulfovibrio_vulgaris__Miyazaki_F__uid59089

Desulfurispirillum_indicum_S5_uid45897

Desulfurivibrio_alkaliphilus_AHT2_uid49487

Desulfurobacterium_thermolithotrophum_DSM_11699_uid63405

Desulfurococcus_fermentans_DSM_16532_uid75119

Desulfurococcus_kamchatkensis_1221n_uid59133

Desulfurococcus_mucosus_DSM_2162_uid62227

Dichelobacter_nodosus_VCS1703A_uid57643

Dickeya_dadantii_3937_uid52537

Dickeya_dadantii_Ech586_uid42519

Dickeya_dadantii_Ech703_uid59363

Dickeya_zeae_Ech1591_uid59297

Dictyoglomus_thermophilum_H_6_12_uid59439

Dictyoglomus_turgidum_DSM_6724_uid59177

Dinoroseobacter_shibae_DFL_12_uid58707

Dyadobacter_fermentans_DSM_18053_uid59049

Edwardsiella_ictaluri_93_146_uid59403

Edwardsiella_tarda_EIB202_uid41819

Edwardsiella_tarda_FL6_60_uid159657

Eggerthella_YY7918_uid68707

Eggerthella_lenta_DSM_2243_uid59079

Ehrlichia_canis_Jake_uid58071

Ehrlichia_chaffeensis_Arkansas_uid57933

Ehrlichia_ruminantium_Gardel_uid58245

Ehrlichia_ruminantium_Welgevonden_uid58013

Ehrlichia_ruminantium_Welgevonden_uid58243

Elusimicrobium_minutum_Pei191_uid58949

Emticicia_oligotrophica_DSM_17448_uid177079

Enterobacter_638_uid58727

Enterobacter_aerogenes_KCTC_2190_uid68103

Enterobacter_asburiae_LF7a_uid72793

Enterobacter_cloacae_ATCC_13047_uid48363

Enterobacter_cloacae_EcWSU1_uid80739

Enterobacter_cloacae_SCF1_uid59969

Enterobacter_cloacae_dissolvens_SDM_uid168997

Enterococcus_faecalis_62_uid159663

Enterococcus_faecalis_D32_uid171261

Enterococcus_faecalis_OG1RF_uid54927

Enterococcus_faecalis_V583_uid57669

Enterococcus_faecium_Aus0004_uid87025

Enterococcus_faecium_DO_uid55353

Enterococcus_hirae_ATCC_9790_uid70619

Erwinia_Ejp617_uid159955

Erwinia_amylovora_ATCC_49946_uid46943

Erwinia_amylovora_CFBP1430_uid46839

Erwinia_billingiae_Eb661_uid50547

Erwinia_pyrifoliae_DSM_12163_uid159693

Erwinia_pyrifoliae_Ep1_96_uid40659

Erwinia_tasmaniensis_Et1_99_uid59029

Erysipelothrix_rhusiopathiae_Fujisawa_uid68021

Erythrobacter_litoralis_HTCC2594_uid58299

Escherichia_blattae_DSM_4481_uid165043

Escherichia_coli_042_uid161985

Escherichia_coli_536_uid58531

Escherichia_coli_55989_uid59383

Escherichia_coli_ABU_83972_uid161975

Escherichia_coli_APEC_O1_uid58623

Escherichia_coli_ATCC_8739_uid58783

Escherichia_coli_BL21_DE3__uid161947

Escherichia_coli_BL21_DE3__uid161949

Escherichia_coli_BW2952_uid59391

Escherichia_coli_B_REL606_uid58803

Escherichia_coli_CFT073_uid57915

Escherichia_coli_DH1_uid161951

Escherichia_coli_DH1_uid162051

Escherichia_coli_E24377A_uid58395

Escherichia_coli_ED1a_uid59379

Escherichia_coli_ETEC_H10407_uid161993

Escherichia_coli_HS_uid58393

Escherichia_coli_IAI1_uid59377

Escherichia_coli_IAI39_uid59381

Escherichia_coli_IHE3034_uid162007

Escherichia_coli_KO11FL_uid162099

Escherichia_coli_KO11FL_uid52593

Escherichia_coli_K_12_substr__DH10B_uid58979

Escherichia_coli_K_12_substr__MG1655_uid57779

Escherichia_coli_K_12_substr__W3110_uid161931

Escherichia_coli_LF82_uid161965

Escherichia_coli_NA114_uid162139

Escherichia_coli_O103_H2_12009_uid41013

Escherichia_coli_O104_H4_2009EL_2071_uid176128

Escherichia_coli_O104_H4_2011C_3493_uid176127

Escherichia_coli_O111_H__11128_uid41023

Escherichia_coli_O127_H6_E2348_69_uid59343

Escherichia_coli_O157_H7_EC4115_uid59091

Escherichia_coli_O157_H7_EDL933_uid57831

Escherichia_coli_O157_H7_Sakai_uid57781

Escherichia_coli_O157_H7_TW14359_uid59235

Escherichia_coli_O26_H11_11368_uid41021

Escherichia_coli_O55_H7_CB9615_uid46655

Escherichia_coli_O55_H7_RM12579_uid162153

Escherichia_coli_O7_K1_CE10_uid162115

Escherichia_coli_O83_H1_NRG_857C_uid161987

Escherichia_coli_P12b_uid162061

Escherichia_coli_S88_uid62979

Escherichia_coli_SE11_uid59425

Escherichia_coli_SE15_uid161939

Escherichia_coli_SMS_3_5_uid58919

Escherichia_coli_UM146_uid162043

Escherichia_coli_UMN026_uid62981

Escherichia_coli_UMNK88_uid161991

Escherichia_coli_UTI89_uid58541

Escherichia_coli_W_uid162011

Escherichia_coli_W_uid162101

Escherichia_coli_Xuzhou21_uid163995

Escherichia_coli__BL21_Gold_DE3_pLysS_AG__uid59245

Escherichia_coli__clone_D_i14__uid162049

Escherichia_coli__clone_D_i2__uid162047

Escherichia_fergusonii_ATCC_35469_uid59375

Ethanoligenens_harbinense_YUAN_3_uid46255

Eubacterium_eligens_ATCC_27750_uid59171

Eubacterium_limosum_KIST612_uid59777

Eubacterium_rectale_ATCC_33656_uid59169

Exiguobacterium_AT1b_uid59093

Exiguobacterium_antarcticum_B7_uid176125

Exiguobacterium_sibiricum_255_15_uid58053

Ferrimonas_balearica_DSM_9799_uid53371

Ferroglobus_placidus_DSM_10642_uid40863

Fervidicoccus_fontis_Kam940_uid162201

Fervidobacterium_nodosum_Rt17_B1_uid58625

Fervidobacterium_pennivorans_DSM_9078_uid78143

Fibrobacter_succinogenes_S85_uid161919

Fibrobacter_succinogenes_S85_uid41169

Filifactor_alocis_ATCC_35896_uid46625

Finegoldia_magna_ATCC_29328_uid58867

Flavobacteriaceae_bacterium_3519_10_uid59413

Flavobacteriales_bacterium_HTCC2170_uid51877

Flavobacterium_branchiophilum_FL_15_uid73421

Flavobacterium_columnare_ATCC_49512_uid80731

Flavobacterium_indicum_GPTSA100_9_uid157999

Flavobacterium_johnsoniae_UW101_uid58493

Flavobacterium_psychrophilum_JIP02_86_uid61627

Flexibacter_litoralis_DSM_6794_uid168257

Flexistipes_sinusarabici_DSM_4947_uid68147

Fluviicola_taffensis_DSM_16823_uid65271

Francisella_TX077308_uid68321

Francisella_cf__novicida_3523_uid162107

Francisella_cf__novicida_Fx1_uid162105

Francisella_noatunensis_orientalis_Toba_04_uid164779

Francisella_novicida_U112_uid58499

Francisella_philomiragia_ATCC_25017_uid59105

Francisella_tularensis_FSC198_uid58693

Francisella_tularensis_NE061598_uid161973

Francisella_tularensis_SCHU_S4_uid57589

Francisella_tularensis_TI0902_uid89373

Francisella_tularensis_TIGB03_uid89379

Francisella_tularensis_WY96_3418_uid58811

Francisella_tularensis_holarctica_FTNF002_00_uid58999

Francisella_tularensis_holarctica_LVS_uid58595

Francisella_tularensis_holarctica_OSU18_uid58687

Francisella_tularensis_mediasiatica_FSC147_uid58939

Frankia_CcI3_uid58397

Frankia_EAN1pec_uid58367

Frankia_EuI1c_uid42615

Frankia_alni_ACN14a_uid58695

Frankia_symbiont_of_Datisca_glomerata_uid46257

Frateuria_aurantia_DSM_6220_uid81775

Fusobacterium_nucleatum_ATCC_25586_uid57885

Gallibacterium_anatis_UMN179_uid66567

Gallionella_capsiferriformans_ES_2_uid51505

Gardnerella_vaginalis_409_05_uid43211

Gardnerella_vaginalis_ATCC_14019_uid55487

Gardnerella_vaginalis_HMP9231_uid162045

Gemmatimonas_aurantiaca_T_27_uid58813

Geobacillus_C56_T3_uid49467

Geobacillus_WCH70_uid59045

Geobacillus_Y412MC52_uid55381

Geobacillus_Y412MC61_uid41171

Geobacillus_Y4_1MC1_uid55779

Geobacillus_kaustophilus_HTA426_uid58227

Geobacillus_thermodenitrificans_NG80_2_uid58829

Geobacillus_thermoglucosidasius_C56_YS93_uid48129

Geobacillus_thermoleovorans_CCB_US3_UF5_uid82949

Geobacter_FRC_32_uid58543

Geobacter_M18_uid55771

Geobacter_M21_uid59037

Geobacter_bemidjiensis_Bem_uid58749

Geobacter_lovleyi_SZ_uid58713

Geobacter_metallireducens_GS_15_uid57731

Geobacter_sulfurreducens_KN400_uid161977

Geobacter_sulfurreducens_PCA_uid57743

Geobacter_uraniireducens_Rf4_uid58475

Geodermatophilus_obscurus_DSM_43160_uid43725

Glaciecola_4H_3_7_YE_5_uid66595

Glaciecola_nitratireducens_FR1064_uid73759

Gloeobacter_violaceus_PCC_7421_uid58011

Gluconacetobacter_diazotrophicus_PAl_5_uid59075

Gluconacetobacter_diazotrophicus_PAl_5_uid61587

Gluconacetobacter_xylinus_NBRC_3288_uid46523

Gluconobacter_oxydans_621H_uid58239

Gordonia_KTR9_uid174812

Gordonia_bronchialis_DSM_43247_uid41403

Gordonia_polyisoprenivorans_VH2_uid86651

Gramella_forsetii_KT0803_uid58881

Granulibacter_bethesdensis_CGDNIH1_uid58661

Granulicella_mallensis_MP5ACTX8_uid49957

Haemophilus_ducreyi_35000HP_uid57625

Haemophilus_influenzae_10810_uid86647

Haemophilus_influenzae_86_028NP_uid58093

Haemophilus_influenzae_F3031_uid62123

Haemophilus_influenzae_F3047_uid62097

Haemophilus_influenzae_PittEE_uid58591

Haemophilus_influenzae_PittGG_uid58593

Haemophilus_influenzae_R2846_uid161921

Haemophilus_influenzae_R2866_uid161923

Haemophilus_influenzae_Rd_KW20_uid57771

Haemophilus_parainfluenzae_T3T1_uid72801

Haemophilus_parasuis_SH0165_uid59273

Haemophilus_somnus_129PT_uid57929

Haemophilus_somnus_2336_uid57979

Hahella_chejuensis_KCTC_2396_uid58483

Halalkalicoccus_jeotgali_B3_uid50305

Halanaerobium_hydrogeniformans_uid60191

Halanaerobium_praevalens_DSM_2228_uid161959

Haliangium_ochraceum_DSM_14365_uid41425

Haliscomenobacter_hydrossis_DSM_1100_uid66777

Haloarcula_hispanica_ATCC_33960_uid72475

Haloarcula_marismortui_ATCC_43049_uid57719

Halobacillus_halophilus_DSM_2266_uid162033

Halobacterium_NRC_1_uid57769

Halobacterium_salinarum_R1_uid61571

Haloferax_mediterranei_ATCC_33500_uid167315

Haloferax_volcanii_DS2_uid46845

Halogeometricum_borinquense_DSM_11551_uid54919

Halomicrobium_mukohataei_DSM_12286_uid59107

Halomonas_elongata_DSM_2581_uid52781

Halopiger_xanaduensis_SH_6_uid68105

Haloquadratum_walsbyi_C23_uid162019

Haloquadratum_walsbyi_DSM_16790_uid58673

Halorhabdus_utahensis_DSM_12940_uid59189

Halorhodospira_halophila_SL1_uid58473

Halorubrum_lacusprofundi_ATCC_49239_uid58807

Haloterrigena_turkmenica_DSM_5511_uid43501

Halothermothrix_orenii_H_168_uid58585

Halothiobacillus_neapolitanus_c2_uid41317

Helicobacter_acinonychis_Sheeba_uid58685

Helicobacter_bizzozeronii_CIII_1_uid68141

Helicobacter_cetorum_MIT_00_7128_uid162217

Helicobacter_cetorum_MIT_99_5656_uid162215

Helicobacter_cinaedi_PAGU611_uid162219

Helicobacter_felis_ATCC_49179_uid61409

Helicobacter_hepaticus_ATCC_51449_uid57737

Helicobacter_mustelae_12198_uid46647

Helicobacter_pylori_2017_uid161151

Helicobacter_pylori_2018_uid161159

Helicobacter_pylori_26695_uid57787

Helicobacter_pylori_35A_uid49903

Helicobacter_pylori_51_uid161925

Helicobacter_pylori_83_uid161153

Helicobacter_pylori_908_uid159985

Helicobacter_pylori_B38_uid59415

Helicobacter_pylori_B8_uid49873

Helicobacter_pylori_Cuz20_uid159987

Helicobacter_pylori_ELS37_uid158157

Helicobacter_pylori_F16_uid161145

Helicobacter_pylori_F30_uid159991

Helicobacter_pylori_F32_uid161139

Helicobacter_pylori_F57_uid161143

Helicobacter_pylori_G27_uid59305

Helicobacter_pylori_Gambia94_24_uid159493

Helicobacter_pylori_HPAG1_uid58517

Helicobacter_pylori_HUP_B14_uid162213

Helicobacter_pylori_India7_uid161149

Helicobacter_pylori_J99_uid57789

Helicobacter_pylori_Lithuania75_uid159491

Helicobacter_pylori_P12_uid59327

Helicobacter_pylori_PeCan18_uid162211

Helicobacter_pylori_PeCan4_uid53539

Helicobacter_pylori_Puno120_uid159611

Helicobacter_pylori_Puno135_uid161157

Helicobacter_pylori_SJM180_uid53541

Helicobacter_pylori_SNT49_uid159615

Helicobacter_pylori_Sat464_uid159467

Helicobacter_pylori_Shi112_uid162207

Helicobacter_pylori_Shi169_uid162209

Helicobacter_pylori_Shi417_uid162205

Helicobacter_pylori_Shi470_uid59165

Helicobacter_pylori_SouthAfrica7_uid159989

Helicobacter_pylori_XZ274_uid165869

Helicobacter_pylori_uid159983

Helicobacter_pylori_v225d_uid159639

Heliobacterium_modesticaldum_Ice1_uid58279

Herbaspirillum_seropedicae_SmR1_uid50427

Herminiimonas_arsenicoxydans_uid58291

Herpetosiphon_aurantiacus_DSM_785_uid58599

Hippea_maritima_DSM_10411_uid65267

Hirschia_baltica_ATCC_49814_uid59365

Hydrogenobacter_thermophilus_TK_6_uid159875

Hydrogenobacter_thermophilus_TK_6_uid45927

Hydrogenobaculum_Y04AAS1_uid58857

Hyperthermus_butylicus_DSM_5456_uid57755

Hyphomicrobium_MC1_uid68453

Hyphomicrobium_denitrificans_ATCC_51888_uid50325

Hyphomonas_neptunium_ATCC_15444_uid58433

Idiomarina_loihiensis_L2TR_uid58087

Ignavibacterium_album_JCM_16511_uid162097

Ignicoccus_hospitalis_KIN4_I_uid58365

Ignisphaera_aggregans_DSM_17230_uid51875

Ilyobacter_polytropus_DSM_2926_uid59769

Intrasporangium_calvum_DSM_43043_uid61729

Isoptericola_variabilis_225_uid67501

Isosphaera_pallida_ATCC_43644_uid62207

Jannaschia_CCS1_uid58147

Janthinobacterium_Marseille_uid58603

Jonesia_denitrificans_DSM_20603_uid59053

Kangiella_koreensis_DSM_16069_uid59209

Ketogulonicigenium_vulgare_Y25_uid59581

Ketogulonigenium_vulgarum_WSH_001_uid161161

Kineococcus_radiotolerans_SRS30216_uid58067

Kitasatospora_setae_KM_6054_uid77027

Klebsiella_oxytoca_E718_uid170256

Klebsiella_oxytoca_KCTC_1686_uid83159

Klebsiella_pneumoniae_1084_uid174151

Klebsiella_pneumoniae_342_uid59145

Klebsiella_pneumoniae_HS11286_uid84387

Klebsiella_pneumoniae_KCTC_2242_uid162147

Klebsiella_pneumoniae_MGH_78578_uid57619

Klebsiella_pneumoniae_NTUH_K2044_uid59073

Klebsiella_variicola_At_22_uid42113

Kocuria_rhizophila_DC2201_uid59099

Kosmotoga_olearia_TBF_19_5_1_uid59205

Kribbella_flavida_DSM_17836_uid43465

Krokinobacter_4H_3_7_5_uid66593

Kyrpidia_tusciae_DSM_2912_uid48361

Kytococcus_sedentarius_DSM_20547_uid59071

Lacinutrix_5H_3_7_4_uid68067

Lactobacillus_acidophilus_30SC_uid63605

Lactobacillus_acidophilus_NCFM_uid57685

Lactobacillus_amylovorus_GRL1118_uid160233

Lactobacillus_amylovorus_GRL_1112_uid61179

Lactobacillus_brevis_ATCC_367_uid57989

Lactobacillus_buchneri_CD034_uid175525

Lactobacillus_buchneri_NRRL_B_30929_uid66205

Lactobacillus_casei_ATCC_334_uid57985

Lactobacillus_casei_BD_II_uid162119

Lactobacillus_casei_BL23_uid59237

Lactobacillus_casei_LC2W_uid162121

Lactobacillus_casei_Zhang_uid50673

Lactobacillus_crispatus_ST1_uid48359

Lactobacillus_delbrueckii_bulgaricus_2038_uid161929

Lactobacillus_delbrueckii_bulgaricus_ATCC_11842_uid58647

Lactobacillus_delbrueckii_bulgaricus_ATCC_BAA_365_uid57987

Lactobacillus_delbrueckii_bulgaricus_ND02_uid60621

Lactobacillus_fermentum_CECT_5716_uid162003

Lactobacillus_fermentum_IFO_3956_uid58865

Lactobacillus_gasseri_ATCC_33323_uid57687

Lactobacillus_helveticus_DPC_4571_uid58761

Lactobacillus_helveticus_H10_uid162017

Lactobacillus_helveticus_R0052_uid174439

Lactobacillus_johnsonii_DPC_6026_uid162057

Lactobacillus_johnsonii_FI9785_uid41735

Lactobacillus_johnsonii_NCC_533_uid58029

Lactobacillus_kefiranofaciens_ZW3_uid67985

Lactobacillus_plantarum_JDM1_uid59361

Lactobacillus_plantarum_ST_III_uid53537

Lactobacillus_plantarum_WCFS1_uid62911

Lactobacillus_reuteri_DSM_20016_uid58471

Lactobacillus_reuteri_JCM_1112_uid58875

Lactobacillus_reuteri_SD2112_uid55357

Lactobacillus_rhamnosus_ATCC_8530_uid162169

Lactobacillus_rhamnosus_GG_uid161983

Lactobacillus_rhamnosus_GG_uid59313

Lactobacillus_rhamnosus_Lc_705_uid59315

Lactobacillus_ruminis_ATCC_27782_uid73417

Lactobacillus_sakei_23K_uid58281

Lactobacillus_salivarius_CECT_5713_uid162005

Lactobacillus_salivarius_UCC118_uid58233

Lactobacillus_sanfranciscensis_TMW_1_1304_uid72937

Lactococcus_garvieae_ATCC_49156_uid73413

Lactococcus_garvieae_Lg2_uid161935

Lactococcus_lactis_CV56_uid160253

Lactococcus_lactis_Il1403_uid57671

Lactococcus_lactis_KF147_uid42831

Lactococcus_lactis_cremoris_A76_uid160937

Lactococcus_lactis_cremoris_MG1363_uid58837

Lactococcus_lactis_cremoris_NZ9000_uid167481

Lactococcus_lactis_cremoris_SK11_uid57983

Laribacter_hongkongensis_HLHK9_uid59265

Lawsonia_intracellularis_PHE_MN1_00_uid61575

Leadbetterella_byssophila_DSM_17132_uid60161

Legionella_longbeachae_NSW150_uid46099

Legionella_pneumophila_2300_99_Alcoy_uid48801

Legionella_pneumophila_ATCC_43290_uid86885

Legionella_pneumophila_Corby_uid58733

Legionella_pneumophila_HL06041035_uid170534

Legionella_pneumophila_Lens_uid58209

Legionella_pneumophila_Lorraine_uid170535

Legionella_pneumophila_Paris_uid58211

Legionella_pneumophila_Philadelphia_1_uid57609

Leifsonia_xyli_CTCB07_uid57759

Leptospira_biflexa_serovar_Patoc__Patoc_1__Ames__uid58511

Leptospira_biflexa_serovar_Patoc__Patoc_1__Paris__uid58993

Leptospira_borgpetersenii_serovar_Hardjo_bovis_JB197_uid58509

Leptospira_borgpetersenii_serovar_Hardjo_bovis_L550_uid58507

Leptospira_interrogans_serovar_Copenhageni_Fiocruz_L1_130_uid58065

Leptospira_interrogans_serovar_Lai_56601_uid57881

Leptospira_interrogans_serovar_Lai_IPAV_uid161957

Leptospirillum_ferrooxidans_C2_3_uid158171

Leptothrix_cholodnii_SP_6_uid58971

Leptotrichia_buccalis_C_1013_b_uid59211

Leuconostoc_C2_uid68743

Leuconostoc_citreum_KM20_uid58481

Leuconostoc_gasicomitatum_LMG_18811_uid50385

Leuconostoc_kimchii_IMSNU_11154_uid48589

Leuconostoc_mesenteroides_ATCC_8293_uid57919

Leuconostoc_mesenteroides_J18_uid84337

Listeria_innocua_Clip11262_uid61567

Listeria_ivanovii_PAM_55_uid73473

Listeria_monocytogenes_07PF0776_uid162185

Listeria_monocytogenes_08_5923_uid43727

Listeria_monocytogenes_10403S_uid54461

Listeria_monocytogenes_ATCC_19117_uid175109

Listeria_monocytogenes_Clip80459_uid59317

Listeria_monocytogenes_EGD_e_uid61583

Listeria_monocytogenes_FSL_R2_561_uid54441

Listeria_monocytogenes_Finland_1998_uid54443

Listeria_monocytogenes_HCC23_uid59203

Listeria_monocytogenes_J0161_uid54459

Listeria_monocytogenes_L312_uid175768

Listeria_monocytogenes_M7_uid162131

Listeria_monocytogenes_SLCC2376_uid175111

Listeria_monocytogenes_SLCC2378_uid175105

Listeria_monocytogenes_SLCC2479_uid175108

Listeria_monocytogenes_SLCC2540_uid175106

Listeria_monocytogenes_SLCC5850_uid175110

Listeria_monocytogenes_SLCC7179_uid175107

Listeria_monocytogenes_serotype_4a_L99_uid161953

Listeria_monocytogenes_serotype_4b_F2365_uid57689

Listeria_monocytogenes_serotype_7_SLCC2482_uid174871

Listeria_monocytogenes_uid43671

Listeria_seeligeri_serovar_1_2b_SLCC3954_uid46215

Listeria_welshimeri_serovar_6b_SLCC5334_uid61605

Lysinibacillus_sphaericus_C3_41_uid58945

Macrococcus_caseolyticus_JCSC5402_uid59003

Magnetococcus_MC_1_uid57833

Magnetospirillum_magneticum_AMB_1_uid58527

Mahella_australiensis_50_1_BON_uid66917

Mannheimia_succiniciproducens_MBEL55E_uid58197

Maricaulis_maris_MCS10_uid58689

Marinithermus_hydrothermalis_DSM_14884_uid65783

Marinitoga_piezophila_KA3_uid81629

Marinobacter_adhaerens_HP15_uid162009

Marinobacter_aquaeolei_VT8_uid59419

Marinobacter_hydrocarbonoclasticus_ATCC_49840_uid162203

Marinomonas_MWYL1_uid58715

Marinomonas_mediterranea_MMB_1_uid64753

Marinomonas_posidonica_IVIA_Po_181_uid67323

Marivirga_tractuosa_DSM_4126_uid60837

Megasphaera_elsdenii_DSM_20460_uid71135

Meiothermus_ruber_DSM_1279_uid46661

Meiothermus_silvanus_DSM_9946_uid49485

Melioribacter_roseus_P3M_uid170941

Melissococcus_plutonius_ATCC_35311_uid66803

Melissococcus_plutonius_DAT561_uid89371

Mesoplasma_florum_L1_uid58055

Mesorhizobium_ciceri_biovar_biserrulae_WSM1271_uid62101

Mesorhizobium_loti_MAFF303099_uid57601

Mesorhizobium_opportunistum_WSM2075_uid40861

Mesotoga_prima_MesG1_Ag_4_2_uid52599

Metallosphaera_cuprina_Ar_4_uid66329

Metallosphaera_sedula_DSM_5348_uid58717

Methanobacterium_AL_21_uid63623

Methanobacterium_SWAN_1_uid67359

Methanobrevibacter_ruminantium_M1_uid45857

Methanobrevibacter_smithii_ATCC_35061_uid58827

Methanocaldococcus_FS406_22_uid42499

Methanocaldococcus_fervens_AG86_uid59347

Methanocaldococcus_infernus_ME_uid48803

Methanocaldococcus_jannaschii_DSM_2661_uid57713

Methanocaldococcus_vulcanius_M7_uid41131

Methanocella_arvoryzae_MRE50_uid61623

Methanocella_conradii_HZ254_uid157911

Methanocella_paludicola_SANAE_uid42887

Methanococcoides_burtonii_DSM_6242_uid58023

Methanococcus_aeolicus_Nankai_3_uid58823

Methanococcus_maripaludis_C5_uid58741

Methanococcus_maripaludis_C6_uid58947

Methanococcus_maripaludis_C7_uid58847

Methanococcus_maripaludis_S2_uid58035

Methanococcus_maripaludis_X1_uid70729

Methanococcus_vannielii_SB_uid58767

Methanococcus_voltae_A3_uid49529

Methanocorpusculum_labreanum_Z_uid58785

Methanoculleus_bourgensis_MS2_uid171377

Methanoculleus_marisnigri_JR1_uid58561

Methanohalobium_evestigatum_Z_7303_uid49857

Methanohalophilus_mahii_DSM_5219_uid47313

Methanolobus_psychrophilus_R15_uid177925

Methanoplanus_petrolearius_DSM_11571_uid52695

Methanopyrus_kandleri_AV19_uid57883

Methanoregula_boonei_6A8_uid58815

Methanosaeta_concilii_GP6_uid66207

Methanosaeta_harundinacea_6Ac_uid81199

Methanosaeta_thermophila_PT_uid58469

Methanosalsum_zhilinae_DSM_4017_uid68249

Methanosarcina_acetivorans_C2A_uid57879

Methanosarcina_barkeri_Fusaro_uid57715

Methanosarcina_mazei_Go1_uid57893

Methanosphaera_stadtmanae_DSM_3091_uid58407

Methanosphaerula_palustris_E1_9c_uid59193

Methanospirillum_hungatei_JF_1_uid58181

Methanothermobacter_marburgensis_Marburg_uid51637

Methanothermobacter_thermautotrophicus_Delta_H_uid57877

Methanothermococcus_okinawensis_IH1_uid51535

Methanothermus_fervidus_DSM_2088_uid60167

Methanotorris_igneus_Kol_5_uid67321

Methylacidiphilum_infernorum_V4_uid59161

Methylibium_petroleiphilum_PM1_uid58085

Methylobacillus_flagellatus_KT_uid58049

Methylobacterium_4_46_uid58843

Methylobacterium_chloromethanicum_CM4_uid58933

Methylobacterium_extorquens_AM1_uid57605

Methylobacterium_extorquens_DM4_uid61617

Methylobacterium_extorquens_PA1_uid58821

Methylobacterium_nodulans_ORS_2060_uid59023

Methylobacterium_populi_BJ001_uid58937

Methylobacterium_radiotolerans_JCM_2831_uid58845

Methylocella_silvestris_BL2_uid59433

Methylococcus_capsulatus_Bath_uid57607

Methylomicrobium_alcaliphilum_uid77119

Methylomonas_methanica_MC09_uid67363

Methylophaga_JAM1_uid162947

Methylophaga_JAM7_uid162949

Methylotenera_301_uid49469

Methylotenera_mobilis_JLW8_uid59373

Methylovorus_MP688_uid60723

Methylovorus_glucosetrophus_SIP3_4_uid59367

Micavibrio_aeruginosavorus_ARL_13_uid73585

Microbacterium_testaceum_StLB037_uid62789

Micrococcus_luteus_NCTC_2665_uid59033

Microcystis_aeruginosa_NIES_843_uid59101

Microlunatus_phosphovorus_NM_1_uid68055

Micromonospora_L5_uid45895

Micromonospora_aurantiaca_ATCC_27029_uid42501

Mobiluncus_curtisii_ATCC_43063_uid49695

Modestobacter_marinus_uid167487

Moorella_thermoacetica_ATCC_39073_uid58051

Moraxella_catarrhalis_RH4_uid48809

Muricauda_ruestringensis_DSM_13258_uid72479

Mycobacterium_JDM601_uid67369

Mycobacterium_JLS_uid58489

Mycobacterium_KMS_uid58491

Mycobacterium_MCS_uid58465

Mycobacterium_MOTT36Y_uid164001

Mycobacterium_abscessus_ATCC_19977_uid61613

Mycobacterium_africanum_GM041182_uid68839

Mycobacterium_avium_104_uid57693

Mycobacterium_avium_paratuberculosis_K_10_uid57699

Mycobacterium_bovis_AF2122_97_uid57695

Mycobacterium_bovis_BCG_Mexico_uid86889

Mycobacterium_bovis_BCG_Pasteur_1173P2_uid58781

Mycobacterium_bovis_BCG_Tokyo_172_uid59281

Mycobacterium_canettii_CIPT_140010059_uid70731

Mycobacterium_chubuense_NBB4_uid168322

Mycobacterium_gilvum_PYR_GCK_uid59421

Mycobacterium_gilvum_Spyr1_uid61403

Mycobacterium_intracellulare_ATCC_13950_uid167994

Mycobacterium_intracellulare_MOTT_02_uid89387

Mycobacterium_intracellulare_MOTT_64_uid89385

Mycobacterium_leprae_Br4923_uid59293

Mycobacterium_leprae_TN_uid57697

Mycobacterium_marinum_M_uid59423

Mycobacterium_massiliense_GO_06_uid170732

Mycobacterium_rhodesiae_NBB3_uid75107

Mycobacterium_smegmatis_MC2_155_uid171958

Mycobacterium_smegmatis_MC2_155_uid57701

Mycobacterium_tuberculosis_CCDC5079_uid161943

Mycobacterium_tuberculosis_CCDC5180_uid161941

Mycobacterium_tuberculosis_CDC1551_uid57775

Mycobacterium_tuberculosis_CTRI_2_uid161997

Mycobacterium_tuberculosis_F11_uid58417

Mycobacterium_tuberculosis_H37Ra_uid58853

Mycobacterium_tuberculosis_H37Rv_uid170532

Mycobacterium_tuberculosis_H37Rv_uid57777

Mycobacterium_tuberculosis_KZN_1435_uid59069

Mycobacterium_tuberculosis_KZN_4207_uid83619

Mycobacterium_tuberculosis_KZN_605_uid54947

Mycobacterium_tuberculosis_RGTB327_uid157907

Mycobacterium_tuberculosis_RGTB423_uid162179

Mycobacterium_tuberculosis_UT205_uid162183

Mycobacterium_ulcerans_Agy99_uid62939

Mycobacterium_vanbaalenii_PYR_1_uid58463

Mycoplasma_agalactiae_PG2_uid61619

Mycoplasma_agalactiae_uid46679

Mycoplasma_arthritidis_158L3_1_uid58005

Mycoplasma_bovis_HB0801_uid168665

Mycoplasma_bovis_Hubei_1_uid68691

Mycoplasma_bovis_PG45_uid60859

Mycoplasma_capricolum_ATCC_27343_uid58525

Mycoplasma_conjunctivae_HRC_581_uid59325

Mycoplasma_crocodyli_MP145_uid47087

Mycoplasma_fermentans_JER_uid53543

Mycoplasma_fermentans_M64_uid62099

Mycoplasma_gallisepticum_F_uid162001

Mycoplasma_gallisepticum_NC06_2006_080_5_2P_uid172629

Mycoplasma_gallisepticum_NC95_13295_2_2P_uid172625

Mycoplasma_gallisepticum_NY01_2001_047_5_1P_uid172627

Mycoplasma_gallisepticum_R_high__uid161999

Mycoplasma_gallisepticum_R_low__uid57993

Mycoplasma_gallisepticum_VA94_7994_1_7P_uid172624

Mycoplasma_gallisepticum_WI01_2001_043_13_2P_uid172628

Mycoplasma_genitalium_G37_uid57707

Mycoplasma_genitalium_M2288_uid173372

Mycoplasma_genitalium_M2321_uid173373

Mycoplasma_genitalium_M6282_uid173371

Mycoplasma_genitalium_M6320_uid173370

Mycoplasma_haemocanis_Illinois_uid82367

Mycoplasma_haemofelis_Langford_1_uid62461

Mycoplasma_haemofelis_Ohio2_uid162029

Mycoplasma_hominis_ATCC_23114_uid41875

Mycoplasma_hyopneumoniae_168_uid162053

Mycoplasma_hyopneumoniae_232_uid58205

Mycoplasma_hyopneumoniae_7448_uid58039

Mycoplasma_hyopneumoniae_J_uid58059

Mycoplasma_hyorhinis_GDL_1_uid87003

Mycoplasma_hyorhinis_HUB_1_uid51695

Mycoplasma_hyorhinis_MCLD_uid162087

Mycoplasma_leachii_99_014_6_uid162031

Mycoplasma_leachii_PG50_uid60849

Mycoplasma_mobile_163K_uid58077

Mycoplasma_mycoides_SC_PG1_uid58031

Mycoplasma_mycoides_capri_LC_95010_uid66189

Mycoplasma_penetrans_HF_2_uid57729

Mycoplasma_pneumoniae_309_uid85495

Mycoplasma_pneumoniae_FH_uid162027

Mycoplasma_pneumoniae_M129_uid57709

Mycoplasma_pulmonis_UAB_CTIP_uid61569

Mycoplasma_putrefaciens_KS1_uid72481

Mycoplasma_suis_Illinois_uid61897

Mycoplasma_suis_KI3806_uid63665

Mycoplasma_synoviae_53_uid58061

Mycoplasma_wenyonii_Massachusetts_uid170731

Myxococcus_fulvus_HW_1_uid68443

Myxococcus_xanthus_DK_1622_uid58003

Nakamurella_multipartita_DSM_44233_uid59221

Nanoarchaeum_equitans_Kin4_M_uid58009

Natranaerobius_thermophilus_JW_NM_WN_LF_uid59001

Natrialba_magadii_ATCC_43099_uid46245

Natrinema_J7_uid171337

Natronomonas_pharaonis_DSM_2160_uid58435

Nautilia_profundicola_AmH_uid59345

Neisseria_gonorrhoeae_FA_1090_uid57611

Neisseria_gonorrhoeae_NCCP11945_uid59191

Neisseria_gonorrhoeae_TCDC_NG08107_uid161097

Neisseria_lactamica_020_06_uid60851

Neisseria_meningitidis_053442_uid58587

Neisseria_meningitidis_8013_uid161967

Neisseria_meningitidis_FAM18_uid57825

Neisseria_meningitidis_G2136_uid162085

Neisseria_meningitidis_H44_76_uid162083

Neisseria_meningitidis_M01_240149_uid162079

Neisseria_meningitidis_M01_240355_uid162075

Neisseria_meningitidis_M04_240196_uid162081

Neisseria_meningitidis_MC58_uid57817

Neisseria_meningitidis_NZ_05_33_uid162077

Neisseria_meningitidis_WUE_2594_uid162093

Neisseria_meningitidis_Z2491_uid57819

Neisseria_meningitidis_alpha14_uid61649

Neisseria_meningitidis_alpha710_uid161971

Neorickettsia_risticii_Illinois_uid58889

Neorickettsia_sennetsu_Miyayama_uid57965

Niastella_koreensis_GR20_10_uid83125

Nitratifractor_salsuginis_DSM_16511_uid62183

Nitratiruptor_SB155_2_uid58861

Nitrobacter_hamburgensis_X14_uid58293

Nitrobacter_winogradskyi_Nb_255_uid58295

Nitrosococcus_halophilus_Nc4_uid46803

Nitrosococcus_oceani_ATCC_19707_uid58403

Nitrosococcus_watsonii_C_113_uid50331

Nitrosomonas_AL212_uid55727

Nitrosomonas_Is79A3_uid68745

Nitrosomonas_europaea_ATCC_19718_uid57647

Nitrosomonas_eutropha_C91_uid58363

Nitrosopumilus_maritimus_SCM1_uid58903

Nitrosospira_multiformis_ATCC_25196_uid58361

Nocardia_brasiliensis_ATCC_700358_uid86913

Nocardia_cyriacigeorgica_GUH_2_uid89395

Nocardia_farcinica_IFM_10152_uid58203

Nocardioides_JS614_uid58149

Nocardiopsis_alba_ATCC_BAA_2165_uid174334

Nocardiopsis_dassonvillei_DSM_43111_uid49483

Nostoc_PCC_7120_uid57803

Nostoc_punctiforme_PCC_73102_uid57767

Novosphingobium_PP1Y_uid67383

Novosphingobium_aromaticivorans_DSM_12444_uid57747

Oceanimonas_GK1_uid81627

Oceanithermus_profundus_DSM_14977_uid60855

Oceanobacillus_iheyensis_HTE831_uid57867

Ochrobactrum_anthropi_ATCC_49188_uid58921

Odoribacter_splanchnicus_DSM_20712_uid63397

Oenococcus_oeni_PSU_1_uid59417

Oligotropha_carboxidovorans_OM4_uid162135

Oligotropha_carboxidovorans_OM5_uid59155

Oligotropha_carboxidovorans_OM5_uid72795

Olsenella_uli_DSM_7084_uid51367

Onion_yellows_phytoplasma_OY_M_uid58015

Opitutus_terrae_PB90_1_uid58965

Orientia_tsutsugamushi_Boryong_uid61621

Orientia_tsutsugamushi_Ikeda_uid58869

Ornithobacterium_rhinotracheale_DSM_15997_uid168256

Oscillibacter_valericigenes_uid73895

Owenweeksia_hongkongensis_DSM_17368_uid82951

Paenibacillus_JDR_2_uid59021

Paenibacillus_Y412MC10_uid41127

Paenibacillus_mucilaginosus_3016_uid89377

Paenibacillus_mucilaginosus_K02_uid162117

Paenibacillus_mucilaginosus_KNP414_uid68311

Paenibacillus_polymyxa_E681_uid53477

Paenibacillus_polymyxa_M1_uid162159

Paenibacillus_polymyxa_SC2_uid59583

Paenibacillus_terrae_HPL_003_uid82371

Paludibacter_propionicigenes_WB4_uid60725

Pantoea_At_9b_uid55845

Pantoea_ananatis_AJ13355_uid162073

Pantoea_ananatis_LMG_20103_uid46807

Pantoea_ananatis_PA13_uid162181

Pantoea_ananatis_uid86861

Pantoea_vagans_C9_1_uid49871

Parabacteroides_distasonis_ATCC_8503_uid58301

Parachlamydia_acanthamoebae_UV7_uid68335

Paracoccus_denitrificans_PD1222_uid58187

Parvibaculum_lavamentivorans_DS_1_uid58739

Parvularcula_bermudensis_HTCC2503_uid51641

Pasteurella_multocida_3480_uid161955

Pasteurella_multocida_36950_uid86887

Pasteurella_multocida_HN06_uid156881

Pasteurella_multocida_Pm70_uid57627

Pectobacterium_atrosepticum_SCRI1043_uid57957

Pectobacterium_carotovorum_PC1_uid59295

Pectobacterium_wasabiae_WPP163_uid41297

Pediococcus_claussenii_ATCC_BAA_344_uid81103

Pediococcus_pentosaceus_ATCC_25745_uid57981

Pedobacter_heparinus_DSM_2366_uid59111

Pedobacter_saltans_DSM_12145_uid61349

Pelagibacterium_halotolerans_B2_uid74393

Pelobacter_carbinolicus_DSM_2380_uid58241

Pelobacter_propionicus_DSM_2379_uid58255

Pelodictyon_phaeoclathratiforme_BU_1_uid58173

Pelotomaculum_thermopropionicum_SI_uid58877

Persephonella_marina_EX_H1_uid58119

Petrotoga_mobilis_SJ95_uid58747

Phaeobacter_gallaeciensis_2_10_uid54715

Phaeobacter_gallaeciensis_DSM_17395_uid54717

Phenylobacterium_zucineum_HLK1_uid58959

Photobacterium_profundum_SS9_uid62923

Photorhabdus_asymbiotica_ATCC_43949_uid59243

Photorhabdus_luminescens_laumondii_TTO1_uid61593

Phycisphaera_mikurensis_NBRC_102666_uid157331

Picrophilus_torridus_DSM_9790_uid58041

Pirellula_staleyi_DSM_6068_uid43209

Planctomyces_brasiliensis_DSM_5305_uid60583

Planctomyces_limnophilus_DSM_3776_uid48643

Polaromonas_JS666_uid58207

Polaromonas_naphthalenivorans_CJ2_uid58273

Polymorphum_gilvum_SL003B_26A1_uid65447

Polynucleobacter_necessarius_STIR1_uid58967

Polynucleobacter_necessarius_asymbioticus_QLW_P1DMWA_1_uid58611

Porphyromonas_asaccharolytica_DSM_20707_uid66603

Porphyromonas_gingivalis_ATCC_33277_uid58879

Porphyromonas_gingivalis_TDC60_uid67407

Porphyromonas_gingivalis_W83_uid57641

Prevotella_denticola_F0289_uid65091

Prevotella_intermedia_17_uid163151

Prevotella_melaninogenica_ATCC_25845_uid51377

Prevotella_ruminicola_23_uid47507

Prochlorococcus_marinus_AS9601_uid58307

Prochlorococcus_marinus_CCMP1375_uid57995

Prochlorococcus_marinus_MIT_9211_uid58309

Prochlorococcus_marinus_MIT_9215_uid58819

Prochlorococcus_marinus_MIT_9301_uid58437

Prochlorococcus_marinus_MIT_9303_uid58305

Prochlorococcus_marinus_MIT_9312_uid58357

Prochlorococcus_marinus_MIT_9313_uid57773

Prochlorococcus_marinus_MIT_9515_uid58313

Prochlorococcus_marinus_NATL1A_uid58423

Prochlorococcus_marinus_NATL2A_uid58359

Prochlorococcus_marinus_pastoris_CCMP1986_uid57761

Propionibacterium_acnes_266_uid162059

Propionibacterium_acnes_6609_uid162137

Propionibacterium_acnes_ATCC_11828_uid162177

Propionibacterium_acnes_KPA171202_uid58101

Propionibacterium_acnes_SK137_uid48071

Propionibacterium_acnes_TypeIA2_P_acn17_uid80735

Propionibacterium_acnes_TypeIA2_P_acn31_uid80733

Propionibacterium_acnes_TypeIA2_P_acn33_uid80745

Propionibacterium_freudenreichii_shermanii_CIRM_BIA1_uid49535

Propionibacterium_propionicum_F0230a_uid170533

Prosthecochloris_aestuarii_DSM_271_uid58151

Proteus_mirabilis_HI4320_uid61599

Providencia_stuartii_MRSN_2154_uid162193

Pseudoalteromonas_SM9913_uid61247

Pseudoalteromonas_atlantica_T6c_uid58283

Pseudoalteromonas_haloplanktis_TAC125_uid58431

Pseudogulbenkiania_NH8B_uid73423

Pseudomonas_ND6_uid167583

Pseudomonas_aeruginosa_DK2_uid168996

Pseudomonas_aeruginosa_LESB58_uid59275

Pseudomonas_aeruginosa_M18_uid162089

Pseudomonas_aeruginosa_NCGM2_S1_uid162173

Pseudomonas_aeruginosa_PA7_uid58627

Pseudomonas_aeruginosa_PAO1_uid57945

Pseudomonas_aeruginosa_UCBPP_PA14_uid57977

Pseudomonas_brassicacearum_NFM421_uid66303

Pseudomonas_entomophila_L48_uid58639

Pseudomonas_fluorescens_A506_uid165185

Pseudomonas_fluorescens_F113_uid87037

Pseudomonas_fluorescens_Pf0_1_uid57591

Pseudomonas_fluorescens_Pf_5_uid57937

Pseudomonas_fluorescens_SBW25_uid158693

Pseudomonas_fulva_12_X_uid67351

Pseudomonas_mendocina_NK_01_uid66299

Pseudomonas_mendocina_ymp_uid58723

Pseudomonas_putida_BIRD_1_uid162055

Pseudomonas_putida_DOT_T1E_uid171260

Pseudomonas_putida_F1_uid58355

Pseudomonas_putida_GB_1_uid58735

Pseudomonas_putida_KT2440_uid57843

Pseudomonas_putida_S16_uid68747

Pseudomonas_putida_W619_uid58651

Pseudomonas_stutzeri_A1501_uid58641

Pseudomonas_stutzeri_ATCC_17588___LMG_11199_uid68749

Pseudomonas_stutzeri_CCUG_29243_uid168379

Pseudomonas_stutzeri_DSM_10701_uid170940

Pseudomonas_stutzeri_DSM_4166_uid162113

Pseudomonas_syringae_B728a_uid57931

Pseudomonas_syringae_phaseolicola_1448A_uid58099

Pseudomonas_syringae_tomato_DC3000_uid57967

Pseudonocardia_dioxanivorans_CB1190_uid65087

Pseudovibrio_FO_BEG1_uid82373

Pseudoxanthomonas_spadix_BD_a59_uid75113

Pseudoxanthomonas_suwonensis_11_1_uid62105

Psychrobacter_PRwf_1_uid58459

Psychrobacter_arcticus_273_4_uid58021

Psychrobacter_cryohalolentis_K5_uid58373

Psychroflexus_torquis_ATCC_700755_uid54205

Psychromonas_ingrahamii_37_uid58521

Pusillimonas_T7_7_uid66391

Pyrobaculum_1860_uid82379

Pyrobaculum_aerophilum_IM2_uid57727

Pyrobaculum_arsenaticum_DSM_13514_uid58409

Pyrobaculum_calidifontis_JCM_11548_uid58787

Pyrobaculum_islandicum_DSM_4184_uid58635

Pyrobaculum_oguniense_TE7_uid84411

Pyrococcus_NA2_uid66551

Pyrococcus_ST04_uid167261

Pyrococcus_abyssi_GE5_uid62903

Pyrococcus_furiosus_COM1_uid169620

Pyrococcus_furiosus_DSM_3638_uid57873

Pyrococcus_horikoshii_OT3_uid57753

Pyrococcus_yayanosii_CH1_uid68281

Pyrolobus_fumarii_1A_uid73415

Rahnella_Y9602_uid62715

Rahnella_aquatilis_CIP_78_65___ATCC_33071_uid86855

Rahnella_aquatilis_HX2_uid158049

Ralstonia_eutropha_H16_uid62925

Ralstonia_eutropha_JMP134_uid58047

Ralstonia_pickettii_12D_uid58859

Ralstonia_pickettii_12J_uid58737

Ralstonia_solanacearum_CFBP2957_uid50545

Ralstonia_solanacearum_GMI1000_uid57593

Ralstonia_solanacearum_PSI07_uid50539

Ralstonia_solanacearum_Po82_uid162133

Ramlibacter_tataouinensis_TTB310_uid68279

Renibacterium_salmoninarum_ATCC_33209_uid58899

Rhizobium_NGR234_uid59081

Rhizobium_etli_CFN_42_uid58377

Rhizobium_etli_CIAT_652_uid59115

Rhizobium_leguminosarum_bv__trifolii_WSM1325_uid58991

Rhizobium_leguminosarum_bv__trifolii_WSM2304_uid58997

Rhizobium_leguminosarum_bv__viciae_3841_uid57955

Rhodobacter_capsulatus_SB_1003_uid47509

Rhodobacter_sphaeroides_2_4_1_uid57653

Rhodobacter_sphaeroides_ATCC_17025_uid58451

Rhodobacter_sphaeroides_ATCC_17029_uid58449

Rhodobacter_sphaeroides_KD131_uid59277

Rhodococcus_equi_103S_uid60171

Rhodococcus_erythropolis_PR4_uid59019

Rhodococcus_jostii_RHA1_uid58325

Rhodococcus_opacus_B4_uid13791

Rhodoferax_ferrireducens_T118_uid58353

Rhodomicrobium_vannielii_ATCC_17100_uid43247

Rhodopirellula_baltica_SH_1_uid61589

Rhodopseudomonas_palustris_BisA53_uid58445

Rhodopseudomonas_palustris_BisB18_uid58443

Rhodopseudomonas_palustris_BisB5_uid58441

Rhodopseudomonas_palustris_CGA009_uid62901

Rhodopseudomonas_palustris_DX_1_uid43327

Rhodopseudomonas_palustris_HaA2_uid58439

Rhodopseudomonas_palustris_TIE_1_uid58995

Rhodospirillum_centenum_SW_uid58805

Rhodospirillum_photometricum_uid159003

Rhodospirillum_rubrum_ATCC_11170_uid57655

Rhodospirillum_rubrum_F11_uid162149

Rhodothermus_marinus_DSM_4252_uid41729

Rhodothermus_marinus_SG0_5JP17_172_uid72767

Rickettsia_africae_ESF_5_uid58799

Rickettsia_akari_Hartford_uid58161

Rickettsia_australis_Cutlack_uid158039

Rickettsia_bellii_OSU_85_389_uid58681

Rickettsia_bellii_RML369_C_uid58405

Rickettsia_canadensis_CA410_uid88063

Rickettsia_canadensis_McKiel_uid58159

Rickettsia_conorii_Malish_7_uid57633

Rickettsia_felis_URRWXCal2_uid58331

Rickettsia_heilongjiangensis_054_uid70839

Rickettsia_japonica_YH_uid73963

Rickettsia_massiliae_AZT80_uid86751

Rickettsia_massiliae_MTU5_uid58801

Rickettsia_montanensis_OSU_85_930_uid158043

Rickettsia_parkeri_Portsmouth_uid158045

Rickettsia_peacockii_Rustic_uid59301

Rickettsia_philipii_364D_uid89383

Rickettsia_prowazekii_BuV67_CWPP_uid158063

Rickettsia_prowazekii_Chernikova_uid158053

Rickettsia_prowazekii_Dachau_uid158057

Rickettsia_prowazekii_GvV257_uid158051

Rickettsia_prowazekii_Katsinyian_uid158055

Rickettsia_prowazekii_Madrid_E_uid61565

Rickettsia_prowazekii_Rp22_uid161945

Rickettsia_prowazekii_RpGvF24_uid158065

Rickettsia_rhipicephali_3_7_female6_CWPP_uid156977

Rickettsia_rickettsii_Arizona_uid86655

Rickettsia_rickettsii_Brazil_uid88069

Rickettsia_rickettsii_Colombia_uid86653

Rickettsia_rickettsii_Hauke_uid86659

Rickettsia_rickettsii_Hino_uid86657

Rickettsia_rickettsii_Hlp_2_uid88067

Rickettsia_rickettsii_Iowa_uid58961

Rickettsia_rickettsii__Sheila_Smith__uid58027

Rickettsia_slovaca_13_B_uid82369

Rickettsia_slovaca_D_CWPP_uid158159

Rickettsia_typhi_B9991CWPP_uid158357

Rickettsia_typhi_TH1527_uid158161

Rickettsia_typhi_Wilmington_uid58063

Riemerella_anatipestifer_ATCC_11845___DSM_15868_uid159857

Riemerella_anatipestifer_ATCC_11845___DSM_15868_uid60727

Riemerella_anatipestifer_RA_GD_uid162013

Robiginitalea_biformata_HTCC2501_uid58285

Roseburia_hominis_A2_183_uid73419

Roseiflexus_RS_1_uid58523

Roseiflexus_castenholzii_DSM_13941_uid58287

Roseobacter_denitrificans_OCh_114_uid58597

Roseobacter_litoralis_Och_149_uid54719

Rothia_dentocariosa_ATCC_17931_uid49331

Rothia_mucilaginosa_uid43093

Rubrivivax_gelatinosus_IL144_uid158163

Rubrobacter_xylanophilus_DSM_9941_uid58057

Ruegeria_TM1040_uid58193

Ruegeria_pomeroyi_DSS_3_uid57863

Ruminococcus_albus_7_uid51721

Runella_slithyformis_DSM_19594_uid68317

Saccharomonospora_viridis_DSM_43017_uid59055

Saccharophagus_degradans_2_40_uid57921

Saccharopolyspora_erythraea_NRRL_2338_uid62947

Salinibacter_ruber_DSM_13855_uid58513

Salinibacter_ruber_M8_uid47323

Salinispora_arenicola_CNS_205_uid58659

Salinispora_tropica_CNB_440_uid58565

Salmonella_bongori_NCTC_12419_uid70155

Salmonella_enterica_arizonae_serovar_62_z4_z23__RSK2980_uid58191

Salmonella_enterica_serovar_Agona_SL483_uid59431

Salmonella_enterica_serovar_Choleraesuis_SC_B67_uid58017

Salmonella_enterica_serovar_Dublin_CT_02021853_uid58917

Salmonella_enterica_serovar_Enteritidis_P125109_uid59247

Salmonella_enterica_serovar_Gallinarum_287_91_uid59249

Salmonella_enterica_serovar_Gallinarum_pullorum_RKS5078_uid87035

Salmonella_enterica_serovar_Heidelberg_B182_uid162195

Salmonella_enterica_serovar_Heidelberg_SL476_uid58973

Salmonella_enterica_serovar_Newport_SL254_uid58831

Salmonella_enterica_serovar_Paratyphi_A_AKU_12601_uid59269

Salmonella_enterica_serovar_Paratyphi_A_ATCC_9150_uid58201

Salmonella_enterica_serovar_Paratyphi_B_SPB7_uid59097

Salmonella_enterica_serovar_Paratyphi_C_RKS4594_uid59063

Salmonella_enterica_serovar_Schwarzengrund_CVM19633_uid58915

Salmonella_enterica_serovar_Typhi_CT18_uid57793

Salmonella_enterica_serovar_Typhi_P_stx_12_uid87001

Salmonella_enterica_serovar_Typhi_Ty2_uid57973

Salmonella_enterica_serovar_Typhimurium_14028S_uid86059

Salmonella_enterica_serovar_Typhimurium_798_uid158047

Salmonella_enterica_serovar_Typhimurium_LT2_uid57799

Salmonella_enterica_serovar_Typhimurium_SL1344_uid86645

Salmonella_enterica_serovar_Typhimurium_ST4_74_uid84393

Salmonella_enterica_serovar_Typhimurium_T000240_uid84397

Salmonella_enterica_serovar_Typhimurium_UK_1_uid87049

Salmonella_enterica_serovar_Typhimurium_uid86061

Salmonella_enterica_serovar_Weltevreden_2007_60_3289_1_uid178014

Sanguibacter_keddieii_DSM_10542_uid40845

Saprospira_grandis_Lewin_uid89375

Sebaldella_termitidis_ATCC_33386_uid41865

Segniliparus_rotundus_DSM_44985_uid49049

Selenomonas_ruminantium_lactilytica_TAM6421_uid157247

Selenomonas_sputigena_ATCC_35185_uid55329

Serratia_AS12_uid67315

Serratia_AS13_uid162065

Serratia_plymuthica_AS9_uid67313

Serratia_proteamaculans_568_uid58725

Serratia_symbiotica__Cinara_cedri__uid82363

Shewanella_ANA_3_uid58347

Shewanella_MR_4_uid58345

Shewanella_MR_7_uid58343

Shewanella_W3_18_1_uid58341

Shewanella_amazonensis_SB2B_uid58257

Shewanella_baltica_BA175_uid52601

Shewanella_baltica_OS117_uid162025

Shewanella_baltica_OS155_uid58259

Shewanella_baltica_OS185_uid58743

Shewanella_baltica_OS195_uid58261

Shewanella_baltica_OS223_uid58775

Shewanella_baltica_OS678_uid50553

Shewanella_denitrificans_OS217_uid58263

Shewanella_frigidimarina_NCIMB_400_uid58265

Shewanella_halifaxensis_HAW_EB4_uid59007

Shewanella_loihica_PV_4_uid58349

Shewanella_oneidensis_MR_1_uid57949

Shewanella_pealeana_ATCC_700345_uid58705

Shewanella_piezotolerans_WP3_uid58745

Shewanella_putrefaciens_200_uid161927

Shewanella_putrefaciens_CN_32_uid58267

Shewanella_sediminis_HAW_EB3_uid58835

Shewanella_violacea_DSS12_uid47085

Shewanella_woodyi_ATCC_51908_uid58721

Shigella_boydii_CDC_3083_94_uid58415

Shigella_boydii_Sb227_uid58215

Shigella_dysenteriae_Sd197_uid58213

Shigella_flexneri_2002017_uid159233

Shigella_flexneri_2a_2457T_uid57991

Shigella_flexneri_2a_301_uid62907

Shigella_flexneri_5_8401_uid58583

Shigella_sonnei_53G_uid84383

Shigella_sonnei_Ss046_uid58217

Sideroxydans_lithotrophicus_ES_1_uid46801

Simiduia_agarivorans_SA1_uid177713

Simkania_negevensis_Z_uid68451

Sinorhizobium_fredii_HH103_uid86865

Sinorhizobium_fredii_USDA_257_uid168059

Sinorhizobium_medicae_WSM419_uid58549

Sinorhizobium_meliloti_1021_uid57603

Sinorhizobium_meliloti_AK83_uid52607

Sinorhizobium_meliloti_BL225C_uid52605

Sinorhizobium_meliloti_SM11_uid159685

Slackia_heliotrinireducens_DSM_20476_uid59051

Sodalis_glossinidius__morsitans__uid58553

Solibacillus_silvestris_StLB046_uid168516

Solitalea_canadensis_DSM_3403_uid81783

Sorangium_cellulosum__So_ce_56__uid61629

Sphaerobacter_thermophilus_DSM_20745_uid41997

Sphaerochaeta_pleomorpha_Grapes_uid82365

Sphingobacterium_21_uid64755

Sphingobium_SYK_6_uid73353

Sphingobium_chlorophenolicum_L_1_uid52597

Sphingobium_japonicum_UT26S_uid47077

Sphingomonas_wittichii_RW1_uid58691

Sphingopyxis_alaskensis_RB2256_uid58351

Spirochaeta_Buddy_uid63633

Spirochaeta_africana_DSM_8902_uid81779

Spirochaeta_caldaria_DSM_7334_uid68753

Spirochaeta_coccoides_DSM_17374_uid66331

Spirochaeta_smaragdinae_DSM_11293_uid51369

Spirochaeta_thermophila_DSM_6192_uid53037

Spirochaeta_thermophila_DSM_6578_uid162041

Spirosoma_linguale_DSM_74_uid43413

Stackebrandtia_nassauensis_DSM_44728_uid46663

Staphylococcus_aureus_04_02981_uid161969

Staphylococcus_aureus_11819_97_uid159981

Staphylococcus_aureus_71193_uid162141

Staphylococcus_aureus_COL_uid57797

Staphylococcus_aureus_ECT_R_2_uid159389

Staphylococcus_aureus_ED133_uid159689

Staphylococcus_aureus_ED98_uid41455

Staphylococcus_aureus_HO_5096_0412_uid162163

Staphylococcus_aureus_JH1_uid58457

Staphylococcus_aureus_JH9_uid58455

Staphylococcus_aureus_JKD6008_uid159855

Staphylococcus_aureus_JKD6159_uid159691

Staphylococcus_aureus_LGA251_uid159391

Staphylococcus_aureus_M013_uid88065

Staphylococcus_aureus_MRSA252_uid57839

Staphylococcus_aureus_MSHR1132_uid89393

Staphylococcus_aureus_MSSA476_uid57841

Staphylococcus_aureus_MW2_uid57903

Staphylococcus_aureus_Mu3_uid58817

Staphylococcus_aureus_Mu50_uid57835

Staphylococcus_aureus_N315_uid57837

Staphylococcus_aureus_NCTC_8325_uid57795

Staphylococcus_aureus_Newman_uid58839

Staphylococcus_aureus_RF122_uid57661

Staphylococcus_aureus_S0385_uid159247

Staphylococcus_aureus_ST398_uid159247

Staphylococcus_aureus_T0131_uid159861

Staphylococcus_aureus_TCH60_uid159859

Staphylococcus_aureus_TW20_uid159241

Staphylococcus_aureus_USA300_FPR3757_uid58555

Staphylococcus_aureus_USA300_TCH1516_uid58925

Staphylococcus_aureus_VC40_uid88071

Staphylococcus_carnosus_TM300_uid59401

Staphylococcus_epidermidis_ATCC_12228_uid57861

Staphylococcus_epidermidis_RP62A_uid57663

Staphylococcus_haemolyticus_JCSC1435_uid62919

Staphylococcus_lugdunensis_HKU09_01_uid46233

Staphylococcus_lugdunensis_N920143_uid162143

Staphylococcus_pseudintermedius_ED99_uid162109

Staphylococcus_pseudintermedius_HKU10_03_uid62125

Staphylococcus_saprophyticus_ATCC_15305_uid58411

Staphylothermus_hellenicus_DSM_12710_uid45893

Staphylothermus_marinus_F1_uid58719

Starkeya_novella_DSM_506_uid48815

Stenotrophomonas_maltophilia_D457_uid162199

Stenotrophomonas_maltophilia_JV3_uid72473

Stenotrophomonas_maltophilia_K279a_uid61647

Stenotrophomonas_maltophilia_R551_3_uid58657

Stigmatella_aurantiaca_DW4_3_1_uid158509

Streptobacillus_moniliformis_DSM_12112_uid41863

Streptococcus_agalactiae_2603V_R_uid57943

Streptococcus_agalactiae_A909_uid57935

Streptococcus_agalactiae_GD201008_001_uid175780

Streptococcus_agalactiae_NEM316_uid61585

Streptococcus_dysgalactiae_equisimilis_ATCC_12394_uid161979

Streptococcus_dysgalactiae_equisimilis_GGS_124_uid59103

Streptococcus_dysgalactiae_equisimilis_RE378_uid176684

Streptococcus_equi_4047_uid59259

Streptococcus_equi_zooepidemicus_ATCC_35246_uid162155

Streptococcus_equi_zooepidemicus_MGCS10565_uid59263

Streptococcus_equi_zooepidemicus_uid59261

Streptococcus_gallolyticus_ATCC_43143_uid162103

Streptococcus_gallolyticus_ATCC_BAA_2069_uid63617

Streptococcus_gallolyticus_UCN34_uid46061

Streptococcus_gordonii_Challis_substr__CH1_uid57667

Streptococcus_infantarius_CJ18_uid87033

Streptococcus_intermedius_JTH08_uid168614

Streptococcus_macedonicus_ACA_DC_198_uid81631

Streptococcus_mitis_B6_uid46097

Streptococcus_mutans_GS_5_uid169223

Streptococcus_mutans_LJ23_uid162197

Streptococcus_mutans_NN2025_uid46353

Streptococcus_mutans_UA159_uid57947

Streptococcus_oralis_Uo5_uid65449

Streptococcus_parasanguinis_ATCC_15912_uid49313

Streptococcus_parasanguinis_FW213_uid163997

Streptococcus_parauberis_KCTC_11537_uid67355

Streptococcus_pasteurianus_ATCC_43144_uid68019

Streptococcus_pneumoniae_670_6B_uid52533

Streptococcus_pneumoniae_70585_uid59125

Streptococcus_pneumoniae_AP200_uid52453

Streptococcus_pneumoniae_ATCC_700669_uid59287

Streptococcus_pneumoniae_CGSP14_uid59181

Streptococcus_pneumoniae_D39_uid58581

Streptococcus_pneumoniae_G54_uid59167

Streptococcus_pneumoniae_Hungary19A_6_uid59117

Streptococcus_pneumoniae_INV104_uid162039

Streptococcus_pneumoniae_INV200_uid162035

Streptococcus_pneumoniae_JJA_uid59121

Streptococcus_pneumoniae_OXC141_uid162037

Streptococcus_pneumoniae_P1031_uid59123

Streptococcus_pneumoniae_R6_uid57859

Streptococcus_pneumoniae_SPNA45_uid174986

Streptococcus_pneumoniae_ST556_uid162191

Streptococcus_pneumoniae_TCH8431_19A_uid49735

Streptococcus_pneumoniae_TIGR4_uid57857

Streptococcus_pneumoniae_Taiwan19F_14_uid59119

Streptococcus_pseudopneumoniae_IS7493_uid71153

Streptococcus_pyogenes_Alab49_uid162171

Streptococcus_pyogenes_M1_GAS_uid57845

Streptococcus_pyogenes_MGAS10270_uid58571

Streptococcus_pyogenes_MGAS10394_uid58105

Streptococcus_pyogenes_MGAS10750_uid58575

Streptococcus_pyogenes_MGAS15252_uid158037

Streptococcus_pyogenes_MGAS1882_uid158061

Streptococcus_pyogenes_MGAS2096_uid58573

Streptococcus_pyogenes_MGAS315_uid57911

Streptococcus_pyogenes_MGAS5005_uid58337

Streptococcus_pyogenes_MGAS6180_uid58335

Streptococcus_pyogenes_MGAS8232_uid57871

Streptococcus_pyogenes_MGAS9429_uid58569

Streptococcus_pyogenes_Manfredo_uid57847

Streptococcus_pyogenes_NZ131_uid59035

Streptococcus_pyogenes_SSI_1_uid57895

Streptococcus_salivarius_57_I_uid162151

Streptococcus_salivarius_CCHSS3_uid70481

Streptococcus_salivarius_JIM8777_uid162145

Streptococcus_sanguinis_SK36_uid58381

Streptococcus_suis_05ZYH33_uid58663

Streptococcus_suis_98HAH33_uid58665

Streptococcus_suis_A7_uid162111

Streptococcus_suis_BM407_uid59321

Streptococcus_suis_D12_uid162127

Streptococcus_suis_D9_uid162125

Streptococcus_suis_GZ1_uid161937

Streptococcus_suis_JS14_uid162095

Streptococcus_suis_P1_7_uid32235

Streptococcus_suis_S735_uid174333

Streptococcus_suis_SC84_uid59323

Streptococcus_suis_SS12_uid162123

Streptococcus_suis_ST1_uid167482

Streptococcus_suis_ST3_uid66327

Streptococcus_thermophilus_CNRZ1066_uid58221

Streptococcus_thermophilus_JIM_8232_uid162157

Streptococcus_thermophilus_LMD_9_uid58327

Streptococcus_thermophilus_LMG_18311_uid58219

Streptococcus_thermophilus_MN_ZLW_002_uid166827

Streptococcus_thermophilus_ND03_uid162015

Streptococcus_uberis_0140J_uid57959

Streptomyces_SirexAA_E_uid72627

Streptomyces_avermitilis_MA_4680_uid57739

Streptomyces_bingchenggensis_BCW_1_uid82931

Streptomyces_cattleya_NRRL_8057___DSM_46488_uid162187

Streptomyces_cattleya_NRRL_8057_uid77117

Streptomyces_coelicolor_A3_2__uid57801

Streptomyces_flavogriseus_ATCC_33331_uid40839

Streptomyces_griseus_NBRC_13350_uid58983

Streptomyces_hygroscopicus_jinggangensis_5008_uid89409

Streptomyces_scabiei_87_22_uid46531

Streptomyces_venezuelae_ATCC_10712_uid177080

Streptomyces_violaceusniger_Tu_4113_uid52609

Streptosporangium_roseum_DSM_43021_uid42521

Sulfobacillus_acidophilus_DSM_10332_uid88061

Sulfobacillus_acidophilus_TPY_uid68841

Sulfolobus_acidocaldarius_DSM_639_uid58379

Sulfolobus_islandicus_HVE10_4_uid162067

Sulfolobus_islandicus_L_D_8_5_uid43679

Sulfolobus_islandicus_L_S_2_15_uid58871

Sulfolobus_islandicus_M_14_25_uid58849

Sulfolobus_islandicus_M_16_27_uid58851

Sulfolobus_islandicus_M_16_4_uid58841

Sulfolobus_islandicus_REY15A_uid162071

Sulfolobus_islandicus_Y_G_57_14_uid58923

Sulfolobus_islandicus_Y_N_15_51_uid58825

Sulfolobus_solfataricus_98_2_uid167998

Sulfolobus_solfataricus_P2_uid57721

Sulfolobus_tokodaii_7_uid57807

Sulfuricurvum_kujiense_DSM_16994_uid60789

Sulfurihydrogenibium_YO3AOP1_uid58855

Sulfurihydrogenibium_azorense_Az_Fu1_uid58121

Sulfurimonas_autotrophica_DSM_16294_uid53043

Sulfurimonas_denitrificans_DSM_1251_uid58185

Sulfurospirillum_barnesii_SES_3_uid168117

Sulfurospirillum_deleyianum_DSM_6946_uid41861

Sulfurovum_NBC37_1_uid58863

Symbiobacterium_thermophilum_IAM_14863_uid58165

Synechococcus_CC9311_uid58123

Synechococcus_CC9605_uid58319

Synechococcus_CC9902_uid58323

Synechococcus_JA_2_3B_a_2_13__uid58537

Synechococcus_JA_3_3Ab_uid58535

Synechococcus_PCC_7002_uid59137

Synechococcus_RCC307_uid61609

Synechococcus_WH_7803_uid61607

Synechococcus_WH_8102_uid61581

Synechococcus_elongatus_PCC_6301_uid58235

Synechococcus_elongatus_PCC_7942_uid58045

Synechocystis_PCC_6803_substr__GT_I_uid158059

Synechocystis_PCC_6803_substr__PCC_N_uid159835

Synechocystis_PCC_6803_substr__PCC_P_uid157913

Synechocystis_PCC_6803_uid159873

Synechocystis_PCC_6803_uid57659

Syntrophobacter_fumaroxidans_MPOB_uid58177

Syntrophobotulus_glycolicus_DSM_8271_uid63343

Syntrophomonas_wolfei_Goettingen_uid58179

Syntrophothermus_lipocalidus_DSM_12680_uid49527

Syntrophus_aciditrophicus_SB_uid58539

Tannerella_forsythia_ATCC_43037_uid83157

Taylorella_asinigenitalis_MCE3_uid73771

Taylorella_equigenitalis_ATCC_35865_uid170255

Taylorella_equigenitalis_MCE9_uid62103

Tepidanaerobacter_Re1_uid66873

Teredinibacter_turnerae_T7901_uid59267

Terriglobus_roseus_DSM_18391_uid168183

Terriglobus_saanensis_SP1PR4_uid53251

Tetragenococcus_halophilus_uid74441

Thauera_MZ1T_uid58987

Thermacetogenium_phaeum_DSM_12270_uid177811

Thermaerobacter_marianensis_DSM_12885_uid61727

Thermanaerovibrio_acidaminovorans_DSM_6589_uid41925

Thermincola_potens_JR_uid48823

Thermoanaerobacter_X513_uid53065

Thermoanaerobacter_X514_uid58589

Thermoanaerobacter_brockii_finnii_Ako_1_uid55639

Thermoanaerobacter_italicus_Ab9_uid46241

Thermoanaerobacter_mathranii_A3_uid49481

Thermoanaerobacter_pseudethanolicus_ATCC_33223_uid58339

Thermoanaerobacter_tengcongensis_MB4_uid57813

Thermoanaerobacter_wiegelii_Rt8_B1_uid52581

Thermoanaerobacterium_saccharolyticum_JW_SL_YS485_uid167781

Thermoanaerobacterium_thermosaccharolyticum_DSM_571_uid51639

Thermoanaerobacterium_xylanolyticum_LX_11_uid63163

Thermobaculum_terrenum_ATCC_BAA_798_uid42011

Thermobifida_fusca_YX_uid57703

Thermobispora_bispora_DSM_43833_uid48999

Thermococcus_4557_uid70841

Thermococcus_AM4_uid54735

Thermococcus_CL1_uid168259

Thermococcus_barophilus_MP_uid54733

Thermococcus_gammatolerans_EJ3_uid59389

Thermococcus_kodakarensis_KOD1_uid58225

Thermococcus_onnurineus_NA1_uid59043

Thermococcus_sibiricus_MM_739_uid59399

Thermocrinis_albus_DSM_14484_uid46231

Thermodesulfatator_indicus_DSM_15286_uid68285

Thermodesulfobacterium_OPB45_uid68283

Thermodesulfobium_narugense_DSM_14796_uid66601

Thermodesulfovibrio_yellowstonii_DSM_11347_uid59257

Thermofilum_pendens_Hrk_5_uid58563

Thermogladius_1633_uid167488

Thermomicrobium_roseum_DSM_5159_uid59341

Thermomonospora_curvata_DSM_43183_uid41885

Thermoplasma_acidophilum_DSM_1728_uid61573

Thermoplasma_volcanium_GSS1_uid57751

Thermoproteus_neutrophilus_V24Sta_uid58421

Thermoproteus_tenax_Kra_1_uid74443

Thermoproteus_uzoniensis_768_20_uid65089

Thermosediminibacter_oceani_DSM_16646_uid51421

Thermosipho_africanus_TCF52B_uid59095

Thermosipho_melanesiensis_BI429_uid58683

Thermosphaera_aggregans_DSM_11486_uid48993

Thermosynechococcus_elongatus_BP_1_uid57907

Thermotoga_RQ2_uid58935

Thermotoga_lettingae_TMO_uid58419

Thermotoga_maritima_MSB8_uid57723

Thermotoga_naphthophila_RKU_10_uid42777

Thermotoga_neapolitana_DSM_4359_uid59065

Thermotoga_petrophila_RKU_1_uid58655

Thermotoga_thermarum_DSM_5069_uid68449

Thermovibrio_ammonificans_HB_1_uid62095

Thermovirga_lienii_DSM_17291_uid77129

Thermus_CCB_US3_UF1_uid81197

Thermus_scotoductus_SA_01_uid62273

Thermus_thermophilus_HB27_uid58033

Thermus_thermophilus_HB8_uid58223

Thermus_thermophilus_JL_18_uid162129

Thermus_thermophilus_SG0_5JP17_16_uid159537

Thioalkalimicrobium_cyclicum_ALM1_uid67391

Thioalkalivibrio_K90mix_uid46181

Thioalkalivibrio_sulfidophilus_HL_EbGr7_uid59179

Thiobacillus_denitrificans_ATCC_25259_uid58189

Thiocystis_violascens_DSM_198_uid74025

Thiomicrospira_crunogena_XCL_2_uid58183

Thiomonas_intermedia_K12_uid48825

Tistrella_mobilis_KA081020_065_uid167486

Tolumonas_auensis_DSM_9187_uid59395

Treponema_azotonutricium_ZAS_9_uid67365

Treponema_brennaborense_DSM_12168_uid66607

Treponema_denticola_ATCC_35405_uid57583

Treponema_pallidum_Chicago_uid159543

Treponema_pallidum_DAL_1_uid87065

Treponema_pallidum_Mexico_A_uid176920

Treponema_pallidum_Nichols_uid57585

Treponema_pallidum_SS14_uid58977

Treponema_pallidum_pertenue_CDC2_uid87051

Treponema_pallidum_pertenue_Gauthier_uid87067

Treponema_pallidum_pertenue_SamoaD_uid87069

Treponema_paraluiscuniculi_Cuniculi_A_uid68447

Treponema_primitia_ZAS_2_uid67367

Treponema_succinifaciens_DSM_2489_uid65781

Trichodesmium_erythraeum_IMS101_uid57925

Tropheryma_whipplei_TW08_27_uid57961

Tropheryma_whipplei_Twist_uid57705

Truepera_radiovictrix_DSM_17093_uid49533

Tsukamurella_paurometabola_DSM_20162_uid48829

Turneriella_parva_DSM_21527_uid168321

Ureaplasma_parvum_serovar_3_ATCC_27815_uid58887

Ureaplasma_parvum_serovar_3_ATCC_700970_uid57711

Ureaplasma_urealyticum_serovar_10_ATCC_33699_uid59011

Variovorax_paradoxus_EPS_uid62107

Variovorax_paradoxus_S110_uid59437

Veillonella_parvula_DSM_2008_uid41927

Verminephrobacter_eiseniae_EF01_2_uid58675

Verrucosispora_maris_AB_18_032_uid66297

Vibrio_EJY3_uid83161

Vibrio_Ex25_uid41601

Vibrio_anguillarum_775_uid68057

Vibrio_cholerae_IEC224_uid89389

Vibrio_cholerae_LMA3984_4_uid159541

Vibrio_cholerae_M66_2_uid59355

Vibrio_cholerae_MJ_1236_uid59387

Vibrio_cholerae_O1_2010EL_1786_uid78933

Vibrio_cholerae_O1_biovar_El_Tor_N16961_uid57623

Vibrio_cholerae_O395_uid159869

Vibrio_cholerae_O395_uid58425

Vibrio_fischeri_ES114_uid58163

Vibrio_fischeri_MJ11_uid58907

Vibrio_furnissii_NCTC_11218_uid82347

Vibrio_harveyi_ATCC_BAA_1116_uid58957

Vibrio_parahaemolyticus_RIMD_2210633_uid57969

Vibrio_splendidus_LGP32_uid59353

Vibrio_vulnificus_CMCP6_uid62909

Vibrio_vulnificus_MO6_24_O_uid62243

Vibrio_vulnificus_YJ016_uid58007

Vulcanisaeta_distributa_DSM_14429_uid52827

Vulcanisaeta_moutnovskia_768_28_uid63631

Waddlia_chondrophila_WSU_86_1044_uid49531

Weeksella_virosa_DSM_16922_uid63627

Weissella_koreensis_KACC_15510_uid68837

Wigglesworthia_glossinidia_endosymbiont_of_Glossina_brevipalpis_uid57853

Wigglesworthia_glossinidia_endosymbiont_of_Glossina_morsitans__Yale_colony__uid88075

Wolbachia_endosymbiont_TRS_of_Brugia_malayi_uid58107

Wolbachia_endosymbiont_of_Culex_quinquefasciatus_Pel_uid61645

Wolbachia_endosymbiont_of_Drosophila_melanogaster_uid57851

Wolbachia_endosymbiont_of_Onchocerca_ochengi_uid171829

Wolbachia_wRi_uid59371

Wolinella_succinogenes_DSM_1740_uid61591

Xanthobacter_autotrophicus_Py2_uid58453

Xanthomonas_albilineans_GPE_PC73_uid43163

Xanthomonas_axonopodis_citri_306_uid57889

Xanthomonas_axonopodis_citrumelo_F1_uid73179

Xanthomonas_campestris_8004_uid57595

Xanthomonas_campestris_ATCC_33913_uid57887

Xanthomonas_campestris_B100_uid61643

Xanthomonas_campestris_raphani_756C_uid159539

Xanthomonas_campestris_vesicatoria_85_10_uid58321

Xanthomonas_oryzae_KACC_10331_uid58155

Xanthomonas_oryzae_MAFF_311018_uid58547

Xanthomonas_oryzae_PXO99A_uid59131

Xanthomonas_oryzae_oryzicola_BLS256_uid54411

Xenorhabdus_bovienii_SS_2004_uid46345

Xenorhabdus_nematophila_ATCC_19061_uid49133

Xylanimonas_cellulosilytica_DSM_15894_uid41935

Xylella_fastidiosa_9a5c_uid57849

Xylella_fastidiosa_GB514_uid162023

Xylella_fastidiosa_M12_uid58763

Xylella_fastidiosa_M23_uid58809

Xylella_fastidiosa_Temecula1_uid57869

Yersinia_enterocolitica_8081_uid57741

Yersinia_enterocolitica_palearctica_105_5R_r__uid63663

Yersinia_enterocolitica_palearctica_Y11_uid162069

Yersinia_pestis_A1122_uid158119

Yersinia_pestis_Angola_uid58485

Yersinia_pestis_Antiqua_uid58607

Yersinia_pestis_CO92_uid57621

Yersinia_pestis_D106004_uid158071

Yersinia_pestis_D182038_uid158073

Yersinia_pestis_KIM_10_uid57875

Yersinia_pestis_Nepal516_uid58609

Yersinia_pestis_Pestoides_F_uid58619

Yersinia_pestis_Z176003_uid47317

Yersinia_pestis_biovar_Medievalis_Harbin_35_uid158537

Yersinia_pestis_biovar_Microtus_91001_uid58037

Yersinia_pseudotuberculosis_IP_31758_uid58487

Yersinia_pseudotuberculosis_IP_32953_uid58157

Yersinia_pseudotuberculosis_PB1__uid59153

Yersinia_pseudotuberculosis_YPIII_uid59151

Zobellia_galactanivorans_uid70621

Zunongwangia_profunda_SM_A87_uid48073

Zymomonas_mobilis_ATCC_10988_uid55403

Zymomonas_mobilis_ATCC_29191_uid170612

Zymomonas_mobilis_NCIMB_11163_uid41019

Zymomonas_mobilis_ZM4_uid58095

Zymomonas_mobilis_pomaceae_ATCC_29192_uid68445

_Cellvibrio__gilvus_ATCC_13127_uid68143

_Nostoc_azollae__0708_uid49725

alpha_proteobacterium_HIMB59_uid175778

alpha_proteobacterium_HIMB5_uid175779

cyanobacterium_UCYN_A_uid43697

gamma_proteobacterium_HdN1_uid51635

halophilic_archaeon_DL31_uid72619

reference_genome_sequence_list

secondary_endosymbiont_of_Ctenarytaina_eucalypti_uid172737

uncultured_Termite_group_1_bacterium_phylotype_Rs_D17_uid59059
