# Supplementary material for: Metagenome fragment classification based on multiple motif-occurrence profiles
Source: PeerJ. 2014 Sep 4;2:e559. doi: 10.7717/peerj.559 (PMC4157293; doi:10.7717/peerj.559)
Supplement: Data S2 — We generated a bacterial BLAST database using makeblastdb (available at: ftp://ftp.ncbi.nlm.nih.gov/blast/executables/blast+/LATEST/) from the blast+package using the option “-in < input file >-dbtype nucl -hasu_index -max_file_sz ‘10GB”’ (the other options were set at the default values). We integrated the 2,112 bacterial reference genomes into a single multifasta format file and used it as the input file. After generating this BLAST database, we ran blastn (available at: ftp://ftp.ncbi.nlm.nih.gov/blast/executables/blast+/LATEST/) from the blast+package using the option “-query < input file >-db < BLAST database >-outfmt 5 -out < output file >” (the other options were set at the default values). We used the top 10,000 fragments of the Sargasso Sea dataset as the input file. [file peerj-02-559-s002.docx]

|  | strain | #fragments |
| --- | --- | --- |
| 1 | *Burkholderia* 383 | 1831 |
| 2 | *Shewanella* ANA 3 | 963 |
| 3 | *Shewanella oneidensis* MR 1 | 312 |
| 4 | *Alpha proteobacterium* HIMB5 | 237 |
| 5 | *Shewanella* MR 7 | 231 |
| 6 | *Shewanella* MR 4 | 206 |
| 7 | *Candidatus Pelagibacter ubique* HTCC1062 | 189 |
| 8 | *Burkholderia cenocepacia* J2315 | 148 |
| 9 | *Aeromonas hydrophila* ATCC 7966 | 123 |
| 10 | *Prochlorococcus marinus* AS9601 | 122 |
| 11 | *Burkholderia cenocepacia* MC0 3 | 119 |
| 12 | *Prochlorococcus marinus* MIT 9301 | 94 |
| 12 | *Burkholderia cenocepacia* HI2424 | 94 |
| 14 | *Synechococcus* WH 8102 | 93 |
| 15 | *Burkholderia phytofirmans* PsJN | 88 |
| 16 | *Burkholderia xenovorans* LB400 | 77 |
| 17 | *Burkholderia ambifaria* AMMD | 68 |
| 18 | *Burkholderia ambifaria* MC40 6 | 57 |
| 19 | *Burkholderia multivorans* ATCC 17616 | 42 |
| 20 | *Burkholderia vietnamiensis* G4 | 41 |
| 21 | *Prochlorococcus marinus* MIT9215 | 40 |
| 22 | *Burkholderia cepacia* GG4 | 36 |
| 23 | *Ralstonia pickettii* 12D | 28 |
| 24 | *Prochlorococcus marinus* MIT9312 | 27 |
| 25 | *Synechococcus* CC9605 | 26 |
| 26 | *Pseudomonas mendocina ymp* | 23 |
| 27 | *Pseudomonas fluorescens* A506 | 21 |
| 27 | *Shewanella putrefaciens* 200 | 21 |
| 29 | *Synechococcus* RCC307 | 20 |
| 30 | *Prochlorococcus marinus pastoris* CCMP1986 | 17 |
| 30 | *Pseudomonas mendocina* NK 01 | 17 |
